# Notes on Shore Flies (Diptera: Ephydridae) from Finland and north-western Russia

**DOI:** 10.3897/BDJ.3.e4701

**Published:** 2015-03-30

**Authors:** Jere Kahanpää, Tadeusz Zatwarnicki

**Affiliations:** ‡ Zoology Unit, Museum of Natural History, University of Helsinki, Helsinki, Finland; § Department of Biosystematics, Opole University, Opole, Poland

**Keywords:** New records, new synonym, *
Trimerina
*, *Scatophila iowana*, faunistics

## Abstract

The recent checklist of the Ephydridae of Finland by Zatwarnicki and Kahanpää (2014) mentioned 13 ephydrid species as new for Finland without further details. This paper presents detailed records for those species and a few other species of interest. Four species are recorded for the first time from Russia. *Trimerina indistincta* Krivosheina, 2004 is herein considered as a new junior synonym of *Trimerina microchaeta* Hendel, 1932, syn. nov.

## Introduction

Finland is an exceptional country in having a record of regularly published checklists of the order Diptera, including shore flies. Three of such lists have been published during the last century: Frey (1941), Hackman (1980) and Kahanpää and Salmela (2014). The Diptera fauna of the country is well studied in comparison to most other countries, but far from perfectly understood.

The most recent list of the Finnish shore fly fauna is Zatwarnicki and Kahanpää (2014) in Kahanpää and Salmela (2014). The Finnish Ephydridae have attracted relatively little attention: few Finnish dipterologists have studied them and no coordinated study of the fauna has been made. The Finnish Museum of Natural History in Helsinki (MZH) holds by far the largest ephydrid collection in the country. Existing faunal works are essentially based on museum material collected between the years 1900 and 1960, mostly by sweep-netting. After a long hiatus, interest in acalyptrate flies resurged in the 21st century among Finnish amateur entomologists. The newer ephydrid material generally remains in private collections.

Zatwarnicki (1997) included a list of the species known from the country by the mid-1990s. Twelve species have been added to the fauna after 1997. Chronologically first is *Notiphila pollinosa* Krivosheina by Krivosheina (1998), then *Calocoenia paurosoma* (Sturtevant & Wheeler) and *Parydra arctica* Clausen by Krivosheina (2000), *Coenia curvicauda* (Meigen) by Haarto and Winqvist (2002), *Gymnoclasiopa pulchella* (Meigen) by Winqvist (2002), *Notiphila major* Stenhammar by Kahanpää and Winqvist (2003), *Trimerina microchaeta* Hendel (as *T. indistincta* Krivosheina) by Krivosheina (2004), *Philotelma defectum* (Haliday) by Mathis et al. (2009), *Atissa pygmaea* (Haliday) by Winqvist (2011), and *Hecamedoides unispinosus* (Collin), *Parydra mitis* (Cresson) and *P. nigritarsis* Strobl by Kahanpää (2013). The identity of the North European *Glenanthe* species was resolved by Zatwarnicki and Mathis (2012).

Thirteen additional species of Ephydridae were recorded for the first time from Finland in the recent checklist (Zatwarnicki and Kahanpää 2014). The checklist format did not allow for the inclusion of record data. This paper provides the previously unpublished records on which the checklist was based. Additionally, one new synonymy is proposed: *Trimerina indistincta* Krivosheina, 2004 is treated as a junior synonym of *Trimerina microchaeta* Hendel, 1932 (syn. nov.). After inclusion of all additions and corrections to the fauna, the number of shore fly species recorded from Finland is now 112.

Some material from north-western Russia, adjacent to the Russian-Finnish border, was also examined during the preparation of the Finnish checklist. Four species are recorded from Russia for the first time.

## Materials and methods

All the specimens discussed in this paper are deposited at the Zoological Museum of the Finnish Museum of Natural History in Helsinki (MZH) unless otherwise specified. Most MZH specimens have unique specimen identifiers in the form of a URL (Uniform Resource Locator). The URL can be used as a web address: it points to a web page with details of the sample in question. While we cannot guarantee that the web content is available in the future, the address (URL) will remain unique. Standard abbreviations are used for the Finnish biogeographical provinces; see the Fauna Entomologica Scandinavica book series or Heikinheimo and Raatikainen (1971) and Heikinheimo and Raatikainen (1981) for details and maps.

Finnish insect collectors usually use a national uniform grid system (*yhtenäiskoordinaatisto*, YKJ) for mapping and labeling purposes. The Darwin Core (DC) field "verbatimCoordinates" is used for the YKJ coordinates printed on labels. The DC fields "decimalLatitude" and "decimalLongitude" in the Taxon treatments section are given in World Geodetic System 1984 (WGS84) decimal degrees. Where no collector-supplied coordinates were available, the records were georeferenced by the senior author on the basis of the collecting locality.

The identity of all included species is based on the study of primary types conducted by the junior author, except for *Hydrellia tarsata* Haliday and *Scatophila mesogramma* (Loew), for which interpretations are based on Collin (1966) and Sturtevant and Wheeler (1953) respectively.

The order of species follows Zatwarnicki and Kahanpää (2014).

## Taxon treatments

### Trimerina microchaeta

Hendel, 1932

Trimerina microchaeta Hendel 1932: 11
= Trimerina indistincta Krivosheina 2004: 631 (syn. nov.)

#### Materials

- **Type status:** Other material. **Occurrence:** catalogNumber: JKA12-0653; recordedBy: Frey, Richard; individualCount: 1; sex: M; lifeStage: adult; associatedSequences: BOLD:2709504; **Taxon:** scientificName: *Trimerina microchaeta* Hendel, 1932; order: Diptera; family: Ephydridae; genus: Trimerina; specificEpithet: microchaeta; scientificNameAuthorship: Hendel, 1932; **Location:** country: Finland; stateProvince: EH; municipality: Hartola; locality: Hirtesalo; verbatimElevation: 80; verbatimCoordinates: 6839:[3]450; decimalLatitude: 61.66; decimalLongitude: 26.07; geodeticDatum: wgs84; georeferencedBy: Kahanpää, Jere; **Identification:** identifiedBy: Kahanpää, Jere; dateIdentified: 2012; **Event:** year: 2002; month: 5; day: 15; habitat: a lake shore; **Record Level:** institutionCode: Coll. J. Kahanpää; basisOfRecord: PreservedSpecimen
- **Type status:** Other material. **Occurrence:** catalogNumber: JKA12-0654; recordedBy: Frey, Richard; individualCount: 1; sex: F; lifeStage: adult; associatedSequences: BOLD:2709505; **Taxon:** scientificName: *Trimerina microchaeta* Hendel, 1932; order: Diptera; family: Ephydridae; genus: Trimerina; specificEpithet: microchaeta; scientificNameAuthorship: Hendel, 1932; **Location:** country: Finland; stateProvince: ES; municipality: Joutsa; locality: Oravakivensalmi; verbatimElevation: 80; verbatimCoordinates: 6845:[3]451; decimalLatitude: 61.71; decimalLongitude: 26.08; geodeticDatum: wgs84; georeferencedBy: Kahanpää, Jere; **Identification:** identifiedBy: Kahanpää, Jere; dateIdentified: 2012; **Event:** year: 2004; month: 6; day: 3; **Record Level:** institutionCode: Coll. J. Kahanpää; basisOfRecord: PreservedSpecimen

#### Distribution

Widespread in Europe; also occurs in the Russian Far East (Kamchatka) (Zatwarnicki 2013).

#### Taxon discussion

After examination of primary types of *Trimerina microchaeta* Hendel and *T. indistincta* Krivosheina we suggest to synonymize these two species. Krivosheina (2004) provided three characters to separate *T. indistincta* from *T. *microchaeta**: a gena-to-eye ratio of 0.17 (versus 0.20 for *T. *microchaeta**), yellow mid and hind coxae (versus all coxae partially dark), mid and hind tibiae entirely yellow (versus mid and hind tibiae with a dark band). These characters separating the two species are not consistent and fall within the variation range of *T. microchaeta*. Both nominal taxa have essentially the same male terminalia with characteristic postgonostyli (=postsurstyli) bearing short and apically rounded antero-ventral processes as illustrated for *T. indistincta* by Krivosheina (2004).

#### Notes

The holotype of *Trimerina indistincta* is shown in Fig. 1a.

### Hydrellia albifrons

Fallén, 1813

#### Materials

- **Type status:** Other material. **Occurrence:** catalogNumber: http://id.luomus.fi/GV.16614; recordedBy: Tiensuu, Lauri; individualCount: 1; sex: M; lifeStage: adult; preparations: gen. prep. on pin; **Taxon:** scientificName: *Hydrellia albifrons* Fallén, 1813; order: Diptera; family: Ephydridae; genus: Hydrellia; specificEpithet: albifrons; scientificNameAuthorship: Fallén, 1813; **Location:** country: Finland; stateProvince: V; municipality: Naantali; decimalLatitude: 60.47; decimalLongitude: 22.47; geodeticDatum: wgs84; coordinateUncertaintyInMeters: 20000; georeferencedBy: Kahanpää, Jere; **Identification:** identifiedBy: Kahanpää, Jere; dateIdentified: 2013-9-26; **Event:** year: 1939; month: 8; day: 16; **Record Level:** institutionCode: MZH; basisOfRecord: PreservedSpecimen
- **Type status:** Other material. **Occurrence:** catalogNumber: http://id.luomus.fi/GV.16615; recordedBy: Tiensuu, Lauri; individualCount: 1; sex: F; lifeStage: adult; **Taxon:** scientificName: *Hydrellia albifrons* Fallén, 1813; order: Diptera; family: Ephydridae; genus: Hydrellia; specificEpithet: albifrons; scientificNameAuthorship: Fallén, 1813; **Location:** country: Finland; stateProvince: V; municipality: Naantali; decimalLatitude: 60.13; decimalLongitude: 22.02; geodeticDatum: wgs84; coordinateUncertaintyInMeters: 20000; georeferencedBy: Kahanpää, Jere; **Identification:** identifiedBy: Kahanpää, Jere; dateIdentified: 2013-9-26; **Event:** year: 1949; month: 7; day: 1; **Record Level:** institutionCode: MZH; basisOfRecord: PreservedSpecimen

#### Diagnosis

Best identified by structures of the male genitalia as figured by Collin (1966) and Canzoneri and Meneghini (1983).

#### Distribution

First recorded from Finland by Zatwarnicki and Kahanpää (2014). Widespread in Europe; also known from North Africa and Pakistan (Zatwarnicki 1988, Zatwarnicki 2013).

#### Ecology

Larvae of this species feed on pondweeds (*Potamogeton*) (Zatwarnicki 1988), the common frogbit (*Hydrocharis morsus-ranae*) (Hering 1926), and possibly also on water plantains (*Alisma plantago-aquatica*) (see Deonier 1971).

### Hydrellia cochleariae

Haliday, 1839

#### Materials

- **Type status:** Other material. **Occurrence:** catalogNumber: http://id.luomus.fi/GV.16690; recordedBy: Palmén, Johan Axel; individualCount: 4; sex: MF; lifeStage: adult; **Taxon:** scientificName: *Hydrellia cochleariae* Haliday, 1839; order: Diptera; family: Ephydridae; genus: Hydrellia; specificEpithet: cochleariae; scientificNameAuthorship: Haliday, 1839; **Location:** country: Finland; stateProvince: U; municipality: Helsinki; locality: Jollas; verbatimLocality: Helsinge; decimalLatitude: 60.16; decimalLongitude: 25.07; geodeticDatum: wgs84; georeferencedBy: Kahanpää, Jere; **Identification:** identifiedBy: Kahanpää, Jere; dateIdentified: 2013-9-26; **Event:** year: 1864; month: 6; day: 20; **Record Level:** institutionCode: MZH; basisOfRecord: PreservedSpecimen
- **Type status:** Other material. **Occurrence:** catalogNumber: http://id.luomus.fi/GV.16691; recordNumber: 4096; recordedBy: Palmén, Johan Axel; individualCount: 5; sex: MF; lifeStage: adult; preparations: gen. prep. on pin; **Taxon:** scientificName: *Hydrellia cochleariae* Haliday, 1839; order: Diptera; family: Ephydridae; genus: Hydrellia; specificEpithet: cochleariae; scientificNameAuthorship: Haliday, 1839; **Location:** country: Finland; stateProvince: U; municipality: Helsinki; locality: Jollas; verbatimLocality: Helsinge; decimalLatitude: 60.16; decimalLongitude: 25.07; geodeticDatum: wgs84; georeferencedBy: Kahanpää, Jere; **Identification:** identifiedBy: Kahanpää, Jere; dateIdentified: 2013-9-26; **Event:** year: 1864; month: 6; day: 20; **Record Level:** institutionCode: MZH; basisOfRecord: PreservedSpecimen
- **Type status:** Other material. **Occurrence:** catalogNumber: http://id.luomus.fi/GV.16690; recordedBy: Meinander, Martin; individualCount: 1; sex: MF; lifeStage: adult; preparations: gen. prep. on pin; **Taxon:** scientificName: *Hydrellia cochleariae* Haliday, 1839; order: Diptera; family: Ephydridae; genus: Hydrellia; specificEpithet: cochleariae; scientificNameAuthorship: Haliday, 1839; **Location:** country: Finland; stateProvince: V; municipality: Korppoo; locality: Jurmo; decimalLatitude: 59.82; decimalLongitude: 21.60; geodeticDatum: wgs84; georeferencedBy: Kahanpää, Jere; **Identification:** identifiedBy: Kahanpää, Jere; dateIdentified: 2013-9-26; **Event:** year: 1960; month: 8; day: 8; **Record Level:** institutionCode: MZH; basisOfRecord: PreservedSpecimen

#### Diagnosis

Best identified by structures of the male genitalia as illustrated by Collin (1966).

#### Distribution

First recorded from Finland by Zatwarnicki and Kahanpää (2014). Central and Western Europe (Zatwarnicki 2013). The Finnish records are the first from Northern Europe.

#### Ecology

Reared from water soldiers (*Stratiotes*), *Potamogeton* (Fischer 1997), and water starworts (*Callitriche*) (Hering 1957). Older, less reliable host records mention *Hydrocharis* as an additional host (see Deonier 1971).

#### Notes

Previously misidentified as *Hydrellia flavicornis* (Fallén, 1823) in Finland (Hackman 1980).

### Hydrellia fulviceps

(Stenhammar, 1844)

#### Materials

- **Type status:** Other material. **Occurrence:** catalogNumber: http://id.luomus.fi/HT.808; recordNumber: 644; recordedBy: Palmén, Johan Axel; individualCount: 1; sex: M; lifeStage: adult; preparations: gen. prep. on pin; **Taxon:** scientificName: *Hydrellia fulvice* ps (Stenhammar, 1844); order: Diptera; family: Ephydridae; genus: Hydrellia; specificEpithet: fulviceps; scientificNameAuthorship: (Stenhammar, 1844); **Location:** country: Finland; stateProvince: A; locality: Åland; verbatimLocality: Aland; decimalLatitude: 60.2; decimalLongitude: 20.0; geodeticDatum: wgs84; coordinateUncertaintyInMeters: 50000; georeferencedBy: Kahanpää, Jere; **Identification:** identifiedBy: Kahanpää, Jere; dateIdentified: 2013-9-26; **Record Level:** institutionCode: MZH; basisOfRecord: PreservedSpecimen

#### Diagnosis

Best identified by structures of the male genitalia as illustrated by Collin (1966).

#### Distribution

First recorded from Finland by Zatwarnicki and Kahanpää (2014). Widespread in Europe, it is recorded also from the Russian Far East (Zatwarnicki 2013).

#### Ecology

No reliable records of the host plant of this species could be found.

#### Notes

Previously misidentified as *Hydrellia chrysostoma* (Meigen, 1830) (sensu Becker 1896) in Finland (Hackman 1980).

### Hydrellia laticeps

(Stenhammar, 1844)

#### Materials

- **Type status:** Other material. **Occurrence:** catalogNumber: http://id.luomus.fi/GV.16624; recordNumber: 272; recordedBy: Frey, Richard; individualCount: 1; sex: M; lifeStage: adult; behavior: sitting on *Juncus*?; **Taxon:** scientificName: *Hydrellia laticeps* (Stenhammar, 1844); order: Diptera; family: Ephydridae; genus: Hydrellia; specificEpithet: laticeps; scientificNameAuthorship: (Stenhammar, 1844); **Location:** country: Finland; stateProvince: U; municipality: Hanko; locality: Tvärminne, Gloet; verbatimLocality: Tvärminne; decimalLatitude: 59.83; decimalLongitude: 23.10; geodeticDatum: wgs84; coordinateUncertaintyInMeters: 1000; georeferencedBy: Kahanpää, Jere; **Identification:** identifiedBy: Kahanpää, Jere; dateIdentified: 2013-9-26; **Event:** year: 1932; month: 7; day: 9; **Record Level:** institutionCode: MZH; basisOfRecord: PreservedSpecimen
- **Type status:** Other material. **Occurrence:** catalogNumber: http://id.luomus.fi/GV.16626; recordNumber: 1533; recordedBy: Frey, Richard; individualCount: 1; sex: M; lifeStage: adult; **Taxon:** scientificName: *Hydrellia laticeps* (Stenhammar, 1844); order: Diptera; family: Ephydridae; genus: Hydrellia; specificEpithet: laticeps; scientificNameAuthorship: (Stenhammar, 1844); **Location:** country: Finland; stateProvince: U; municipality: Hanko; locality: Tvärminne, Gloet; verbatimLocality: Tvärminne; decimalLatitude: 59.83; decimalLongitude: 23.10; geodeticDatum: wgs84; coordinateUncertaintyInMeters: 1000; georeferencedBy: Kahanpää, Jere; **Identification:** identifiedBy: Kahanpää, Jere; dateIdentified: 2013-9-26; **Event:** year: 1932; month: 6; day: 23; **Record Level:** institutionCode: MZH; basisOfRecord: PreservedSpecimen
- **Type status:** Other material. **Occurrence:** catalogNumber: http://id.luomus.fi/GV.16625; recordNumber: 1526; recordedBy: Frey, Richard; individualCount: 1; sex: M; lifeStage: adult; preparations: gen. prep. on pin; **Taxon:** scientificName: *Hydrellia laticeps* (Stenhammar, 1844); order: Diptera; family: Ephydridae; genus: Hydrellia; specificEpithet: laticeps; scientificNameAuthorship: (Stenhammar, 1844); **Location:** country: Finland; stateProvince: U; municipality: Hanko; locality: Tvärminne, Gloet; verbatimLocality: Tvärminne; decimalLatitude: 59.83; decimalLongitude: 23.10; geodeticDatum: wgs84; coordinateUncertaintyInMeters: 1000; georeferencedBy: Kahanpää, Jere; **Identification:** identifiedBy: Kahanpää, Jere; dateIdentified: 2013-9-26; **Event:** year: 1932; month: 6; day: 23; habitat: Phragmites beds; **Record Level:** institutionCode: MZH; basisOfRecord: PreservedSpecimen
- **Type status:** Other material. **Occurrence:** catalogNumber: http://id.luomus.fi/GV.16623; recordNumber: 1534; recordedBy: Frey, Richard; individualCount: 1; sex: M; lifeStage: adult; preparations: gen. prep. on pin; **Taxon:** scientificName: *Hydrellia laticeps* (Stenhammar, 1844); order: Diptera; family: Ephydridae; genus: Hydrellia; specificEpithet: laticeps; scientificNameAuthorship: (Stenhammar, 1844); **Location:** country: Finland; stateProvince: U; municipality: Hanko; locality: Tvärminne, Gloet; verbatimLocality: Tvärminne; decimalLatitude: 59.83; decimalLongitude: 23.10; geodeticDatum: wgs84; coordinateUncertaintyInMeters: 1000; georeferencedBy: Kahanpää, Jere; **Identification:** identifiedBy: Kahanpää, Jere; dateIdentified: 2013-9-26; **Event:** year: 1932; month: 6; day: 23; habitat: Phragmites beds; **Record Level:** institutionCode: MZH; basisOfRecord: PreservedSpecimen
- **Type status:** Other material. **Occurrence:** catalogNumber: http://id.luomus.fi/GV.16630; recordNumber: 1531; recordedBy: Frey, Richard; individualCount: 1; sex: M; lifeStage: adult; **Taxon:** scientificName: *Hydrellia laticeps* (Stenhammar, 1844); order: Diptera; family: Ephydridae; genus: Hydrellia; specificEpithet: laticeps; scientificNameAuthorship: (Stenhammar, 1844); **Location:** country: Finland; stateProvince: U; municipality: Hanko; locality: Tvärminne, Gloet; verbatimLocality: Tvärminne; decimalLatitude: 59.83; decimalLongitude: 23.10; geodeticDatum: wgs84; coordinateUncertaintyInMeters: 1000; georeferencedBy: Kahanpää, Jere; **Identification:** identifiedBy: Kahanpää, Jere; dateIdentified: 2013-9-26; **Event:** year: 1932; month: 6; day: 23; habitat: Phragmites beds; **Record Level:** institutionCode: MZH; basisOfRecord: PreservedSpecimen
- **Type status:** Other material. **Occurrence:** catalogNumber: http://id.luomus.fi/GV.16632; recordNumber: 127; recordedBy: Frey, Richard; individualCount: 1; sex: M; lifeStage: adult; behavior: on Scirpus; **Taxon:** scientificName: *Hydrellia laticeps* (Stenhammar, 1844); order: Diptera; family: Ephydridae; genus: Hydrellia; specificEpithet: laticeps; scientificNameAuthorship: (Stenhammar, 1844); **Location:** country: Finland; stateProvince: U; municipality: Hanko; locality: Tvärminne, Gloet; verbatimLocality: Tvärminne; decimalLatitude: 59.83; decimalLongitude: 23.10; geodeticDatum: wgs84; coordinateUncertaintyInMeters: 1000; georeferencedBy: Kahanpää, Jere; **Identification:** identifiedBy: Kahanpää, Jere; dateIdentified: 2013-9-26; **Event:** year: 1932; month: 7; day: 6; **Record Level:** institutionCode: MZH; basisOfRecord: PreservedSpecimen
- **Type status:** Other material. **Occurrence:** catalogNumber: http://id.luomus.fi/GV.16631; recordNumber: 1496; recordedBy: Frey, Richard; individualCount: 1; sex: M; lifeStage: adult; **Taxon:** scientificName: *Hydrellia laticeps* (Stenhammar, 1844); order: Diptera; family: Ephydridae; genus: Hydrellia; specificEpithet: laticeps; scientificNameAuthorship: (Stenhammar, 1844); **Location:** country: Finland; stateProvince: U; municipality: Hanko; locality: Tvärminne, Gloet; verbatimLocality: Tvärminne; decimalLatitude: 59.83; decimalLongitude: 23.10; geodeticDatum: wgs84; coordinateUncertaintyInMeters: 1000; georeferencedBy: Kahanpää, Jere; **Identification:** identifiedBy: Kahanpää, Jere; dateIdentified: 2013-9-26; **Event:** year: 1932; month: 6; day: 22; habitat: Phragmites beds; **Record Level:** institutionCode: MZH; basisOfRecord: PreservedSpecimen
- **Type status:** Other material. **Occurrence:** catalogNumber: http://id.luomus.fi/GV.16633; recordNumber: 1537; recordedBy: Frey, Richard; individualCount: 1; sex: F; lifeStage: adult; **Taxon:** scientificName: *Hydrellia laticeps* (Stenhammar, 1844); order: Diptera; family: Ephydridae; genus: Hydrellia; specificEpithet: laticeps; scientificNameAuthorship: (Stenhammar, 1844); **Location:** country: Finland; stateProvince: U; municipality: Hanko; locality: Tvärminne, Gloet; verbatimLocality: Tvärminne; decimalLatitude: 59.83; decimalLongitude: 23.10; geodeticDatum: wgs84; coordinateUncertaintyInMeters: 1000; georeferencedBy: Kahanpää, Jere; **Identification:** identifiedBy: Kahanpää, Jere; dateIdentified: 2013-9-26; **Event:** year: 1932; month: 6; day: 23; habitat: Phragmites beds; **Record Level:** institutionCode: MZH; basisOfRecord: PreservedSpecimen
- **Type status:** Other material. **Occurrence:** catalogNumber: http://id.luomus.fi/GV.16634; recordNumber: 1204; recordedBy: Frey, Richard; individualCount: 1; sex: F; lifeStage: adult; **Taxon:** scientificName: *Hydrellia laticeps* (Stenhammar, 1844); order: Diptera; family: Ephydridae; genus: Hydrellia; specificEpithet: laticeps; scientificNameAuthorship: (Stenhammar, 1844); **Location:** country: Finland; stateProvince: U; municipality: Hanko; locality: Tvärminne, Gloet; verbatimLocality: Tvärminne; decimalLatitude: 59.83; decimalLongitude: 23.10; geodeticDatum: wgs84; coordinateUncertaintyInMeters: 1000; georeferencedBy: Kahanpää, Jere; **Identification:** identifiedBy: Kahanpää, Jere; dateIdentified: 2013-9-26; **Event:** year: 1932; month: 8; day: 15; habitat: Phragmites beds; **Record Level:** institutionCode: MZH; basisOfRecord: PreservedSpecimen
- **Type status:** Other material. **Occurrence:** catalogNumber: http://id.luomus.fi/GV.16637; recordNumber: 1197; recordedBy: Frey, Richard; individualCount: 1; sex: F; lifeStage: adult; **Taxon:** scientificName: *Hydrellia laticeps* (Stenhammar, 1844); order: Diptera; family: Ephydridae; genus: Hydrellia; specificEpithet: laticeps; scientificNameAuthorship: (Stenhammar, 1844); **Location:** country: Finland; stateProvince: U; municipality: Hanko; locality: Tvärminne, Gloet; verbatimLocality: Tvärminne; decimalLatitude: 59.83; decimalLongitude: 23.10; geodeticDatum: wgs84; coordinateUncertaintyInMeters: 1000; georeferencedBy: Kahanpää, Jere; **Identification:** identifiedBy: Kahanpää, Jere; dateIdentified: 2013-9-26; **Event:** year: 1932; month: 8; day: 15; **Record Level:** institutionCode: MZH; basisOfRecord: PreservedSpecimen
- **Type status:** Other material. **Occurrence:** catalogNumber: http://id.luomus.fi/GV.16628; recordNumber: 126; recordedBy: Frey, Richard; individualCount: 1; sex: F; lifeStage: adult; behavior: on Scirpus; **Taxon:** scientificName: *Hydrellia laticeps* (Stenhammar, 1844); order: Diptera; family: Ephydridae; genus: Hydrellia; specificEpithet: laticeps; scientificNameAuthorship: (Stenhammar, 1844); **Location:** country: Finland; stateProvince: U; municipality: Hanko; locality: Tvärminne, Gloet; verbatimLocality: Tvärminne; decimalLatitude: 59.83; decimalLongitude: 23.10; geodeticDatum: wgs84; coordinateUncertaintyInMeters: 1000; georeferencedBy: Kahanpää, Jere; **Identification:** identifiedBy: Kahanpää, Jere; dateIdentified: 2013-9-26; **Event:** year: 1932; month: 7; day: 6; **Record Level:** institutionCode: MZH; basisOfRecord: PreservedSpecimen
- **Type status:** Other material. **Occurrence:** catalogNumber: http://id.luomus.fi/GV.16627; recordNumber: 121; recordedBy: Frey, Richard; individualCount: 1; sex: F; lifeStage: adult; behavior: on Scirpus; **Taxon:** scientificName: *Hydrellia laticeps* (Stenhammar, 1844); order: Diptera; family: Ephydridae; genus: Hydrellia; specificEpithet: laticeps; scientificNameAuthorship: (Stenhammar, 1844); **Location:** country: Finland; stateProvince: U; municipality: Hanko; locality: Tvärminne, Gloet; verbatimLocality: Tvärminne; decimalLatitude: 59.83; decimalLongitude: 23.10; geodeticDatum: wgs84; coordinateUncertaintyInMeters: 1000; georeferencedBy: Kahanpää, Jere; **Identification:** identifiedBy: Kahanpää, Jere; dateIdentified: 2013-9-26; **Event:** year: 1932; month: 7; day: 6; **Record Level:** institutionCode: MZH; basisOfRecord: PreservedSpecimen
- **Type status:** Other material. **Occurrence:** catalogNumber: http://id.luomus.fi/GV.16636; recordNumber: 134; recordedBy: Frey, Richard; individualCount: 1; sex: F; lifeStage: adult; behavior: on Scirpus; **Taxon:** scientificName: *Hydrellia laticeps* (Stenhammar, 1844); order: Diptera; family: Ephydridae; genus: Hydrellia; specificEpithet: laticeps; scientificNameAuthorship: (Stenhammar, 1844); **Location:** country: Finland; stateProvince: U; municipality: Hanko; locality: Tvärminne, Gloet; verbatimLocality: Tvärminne; decimalLatitude: 59.83; decimalLongitude: 23.10; geodeticDatum: wgs84; coordinateUncertaintyInMeters: 1000; georeferencedBy: Kahanpää, Jere; **Identification:** identifiedBy: Kahanpää, Jere; dateIdentified: 2013-9-26; **Event:** year: 1932; month: 7; day: 6; **Record Level:** institutionCode: MZH; basisOfRecord: PreservedSpecimen
- **Type status:** Other material. **Occurrence:** catalogNumber: http://id.luomus.fi/GV.16635; recordNumber: 1498; recordedBy: Frey, Richard; individualCount: 1; sex: F; lifeStage: adult; **Taxon:** scientificName: *Hydrellia laticeps* (Stenhammar, 1844); order: Diptera; family: Ephydridae; genus: Hydrellia; specificEpithet: laticeps; scientificNameAuthorship: (Stenhammar, 1844); **Location:** country: Finland; stateProvince: U; municipality: Hanko; locality: Tvärminne, Gloet; verbatimLocality: Tvärminne; decimalLatitude: 59.83; decimalLongitude: 23.10; geodeticDatum: wgs84; coordinateUncertaintyInMeters: 1000; georeferencedBy: Kahanpää, Jere; **Identification:** identifiedBy: Kahanpää, Jere; dateIdentified: 2013-9-26; **Event:** year: 1932; month: 6; day: 22; habitat: Phragmites beds; **Record Level:** institutionCode: MZH; basisOfRecord: PreservedSpecimen
- **Type status:** Other material. **Occurrence:** catalogNumber: http://id.luomus.fi/GV.16629; recordNumber: 269; recordedBy: Frey, Richard; individualCount: 1; sex: F; lifeStage: adult; behavior: on *Juncus*?; **Taxon:** scientificName: *Hydrellia laticeps* (Stenhammar, 1844); order: Diptera; family: Ephydridae; genus: Hydrellia; specificEpithet: laticeps; scientificNameAuthorship: (Stenhammar, 1844); **Location:** country: Finland; stateProvince: U; municipality: Hanko; locality: Tvärminne, Gloet; verbatimLocality: Tvärminne; decimalLatitude: 59.83; decimalLongitude: 23.10; geodeticDatum: wgs84; coordinateUncertaintyInMeters: 1000; georeferencedBy: Kahanpää, Jere; **Identification:** identifiedBy: Kahanpää, Jere; dateIdentified: 2013-9-26; **Event:** year: 1932; month: 7; day: 9; **Record Level:** institutionCode: MZH; basisOfRecord: PreservedSpecimen

#### Diagnosis

Best identified by structures of the male genitalia as illustrated by Collin (1966) and Canzoneri and Meneghini (1983) under the name *Hydrellia flaviceps* (auct. nec Meigen).

#### Distribution

First recorded from Finland by Zatwarnicki and Kahanpää (2014). Widespread in Europe and probably across the Palaearctic Region, but poorly known (Zatwarnicki 2013).

### Hydrellia mutata

(Zetterstedt, 1846)

#### Materials

- **Type status:** Other material. **Occurrence:** catalogNumber: http://id.luomus.fi/GV.16611; recordedBy: Tiensuu, Lauri; individualCount: 1; sex: M; lifeStage: adult; behavior: on Alisma; **Taxon:** scientificName: *Hydrellia mutata* (Zetterstedt, 1846); order: Diptera; family: Ephydridae; genus: Hydrellia; specificEpithet: mutata; scientificNameAuthorship: (Zetterstedt, 1846); **Location:** country: Russia; stateProvince: Carelian Republic; municipality: Sortavala; verbatimLocality: Sortavala; decimalLatitude: 61.7; decimalLongitude: 30.7; geodeticDatum: wgs84; coordinateUncertaintyInMeters: 20000; georeferencedBy: Kahanpää, Jere; **Identification:** identifiedBy: Kahanpää, Jere; dateIdentified: 2013-9-26; **Event:** year: 1936; month: 7; day: 1; verbatimEventDate: 1-VII 36; **Record Level:** institutionCode: MZH; basisOfRecord: PreservedSpecimen
- **Type status:** Other material. **Occurrence:** catalogNumber: http://id.luomus.fi/GV.16612; recordedBy: Tiensuu, Lauri; individualCount: 1; sex: F; lifeStage: adult; behavior: on Alisma; **Taxon:** scientificName: *Hydrellia mutata* (Zetterstedt, 1846); order: Diptera; family: Ephydridae; genus: Hydrellia; specificEpithet: mutata; scientificNameAuthorship: (Zetterstedt, 1846); **Location:** country: Russia; stateProvince: Carelian Republic; municipality: Sortavala; verbatimLocality: Sortavala; decimalLatitude: 61.7; decimalLongitude: 30.7; geodeticDatum: wgs84; coordinateUncertaintyInMeters: 20000; georeferencedBy: Kahanpää, Jere; **Identification:** identifiedBy: Kahanpää, Jere; dateIdentified: 2013-9-26; **Event:** year: 1936; month: 7; day: 1; verbatimEventDate: 1-VII 36; **Record Level:** institutionCode: MZH; basisOfRecord: PreservedSpecimen
- **Type status:** Other material. **Occurrence:** catalogNumber: http://id.luomus.fi/GV.16613; recordedBy: Tiensuu, Lauri; individualCount: 1; sex: F; lifeStage: adult; **Taxon:** scientificName: *Hydrellia mutata* (Zetterstedt, 1846); order: Diptera; family: Ephydridae; genus: Hydrellia; specificEpithet: mutata; scientificNameAuthorship: (Zetterstedt, 1846); **Location:** country: Finland; stateProvince: U; municipality: Helsinki; verbatimLocality: U: Helsinki; decimalLatitude: 60.19; decimalLongitude: 25; geodeticDatum: wgs84; coordinateUncertaintyInMeters: 10000; georeferencedBy: Kahanpää, Jere; **Identification:** identifiedBy: Kahanpää, Jere; dateIdentified: 2013-9-26; **Event:** year: 1958; month: 7; day: 22; verbatimEventDate: 22 VII -58; **Record Level:** institutionCode: MZH; basisOfRecord: PreservedSpecimen
- **Type status:** Other material. **Occurrence:** recordedBy: Kahanpää, Jere; individualCount: 1; sex: M; lifeStage: adult; **Taxon:** scientificName: *Hydrellia mutata* (Zetterstedt, 1846); order: Diptera; family: Ephydridae; genus: Hydrellia; specificEpithet: mutata; scientificNameAuthorship: (Zetterstedt, 1846); **Location:** country: Finland; stateProvince: U; municipality: Helsinki; verbatimLocality: N: Espoo, Kaitalahti; verbatimCoordinates: 66715:33712; verbatimCoordinateSystem: YKJ; decimalLatitude: 60.138; decimalLongitude: 24.691; geodeticDatum: wgs84; coordinateUncertaintyInMeters: 300; georeferencedBy: Kahanpää, Jere; **Identification:** identifiedBy: Kahanpää, Jere; dateIdentified: 2014-02-05; **Event:** year: 2004; month: 8; day: 3; **Record Level:** institutionCode: Coll. Kahanpää; basisOfRecord: PreservedSpecimen
- **Type status:** Other material. **Occurrence:** recordedBy: Kahanpää, Jere; individualCount: 1; sex: M; lifeStage: adult; **Taxon:** scientificName: *Hydrellia mutata* (Zetterstedt, 1846); order: Diptera; family: Ephydridae; genus: Hydrellia; specificEpithet: mutata; scientificNameAuthorship: (Zetterstedt, 1846); **Location:** country: Finland; stateProvince: V; municipality: Lohja; locality: Vaanila; verbatimLocality: Ab: Lohja, Vaanila; verbatimCoordinates: 66919:33409; verbatimCoordinateSystem: YKJ; decimalLatitude: 60.308; decimalLongitude: 24.118; geodeticDatum: wgs84; coordinateUncertaintyInMeters: 50; georeferencedBy: Kahanpää, Jere; **Identification:** identifiedBy: Kahanpää, Jere; dateIdentified: 2014-02-05; **Event:** year: 2004; month: 7; day: 16; **Record Level:** institutionCode: Coll. Kahanpää; basisOfRecord: PreservedSpecimen

#### Diagnosis

Best identified by structures of the male genitalia as illustrated by Collin (1966).

#### Distribution

First recorded from Finland by Zatwarnicki and Kahanpää (2014). Northern, Western and Central Europe (Zatwarnicki 2013).

#### Ecology

Larvae have recently been found feeding on *Alisma* (Robbins 1990) and *Stratiotes*, possibly also *Lemna* and *Alisma* (see Deonier 1971).

#### Notes

Correctly identified by Richard Frey and Lauri Tiensuu but the record was never published. Specimen http://id.luomus.fi/GV.16611 is illustrated in Fig. 1b.

### Hydrellia subalbiceps

Collin, 1966

#### Materials

- **Type status:** Other material. **Occurrence:** catalogNumber: http://id.luomus.fi/GV.16620; recordedBy: Ingelius, Hugo; individualCount: 1; sex: M; lifeStage: adult; preparations: gen. prep. on pin; **Taxon:** scientificName: *Hydrellia subalbiceps* Collin, 1966; order: Diptera; family: Ephydridae; genus: Hydrellia; specificEpithet: subalbiceps; scientificNameAuthorship: Collin, 1966; **Location:** country: Finland; stateProvince: V; municipality: Salo; locality: Uskela; verbatimLocality: Uskela; decimalLatitude: 60.38; decimalLongitude: 23.13; geodeticDatum: wgs84; coordinateUncertaintyInMeters: 20000; georeferencedBy: Kahanpää, Jere; **Identification:** identifiedBy: Kahanpää, Jere; dateIdentified: 2013-9-26; **Record Level:** institutionCode: MZH; basisOfRecord: PreservedSpecimen

#### Diagnosis

Best identified by structures of the male genitalia as illustrated by Collin (1966) and Canzoneri and Meneghini (1983).

#### Distribution

First recorded from Finland by Zatwarnicki and Kahanpää (2014). Widely distributed in Europe and North Africa (Zatwarnicki 2013).

#### Ecology

Larval host plant(s) unknown.

### Hydrellia tarsata

Haliday, 1839

#### Materials

- **Type status:** Other material. **Occurrence:** catalogNumber: http://id.luomus.fi/GV.16707; recordedBy: Tiensuu, Lauri; individualCount: 1; sex: M; lifeStage: adult; behavior: on Stratiotes; preparations: gen. prep. on pin; **Taxon:** scientificName: *Hydrellia tarsata* Haliday, 1839; order: Diptera; family: Ephydridae; genus: Hydrellia; specificEpithet: tarsata; scientificNameAuthorship: Haliday, 1839; **Location:** country: Finland; stateProvince: V; municipality: Naantali; decimalLatitude: 60.13; decimalLongitude: 22.02; geodeticDatum: wgs84; coordinateUncertaintyInMeters: 10000; georeferencedBy: Kahanpää, Jere; **Identification:** identifiedBy: Kahanpää, Jere; dateIdentified: 2013-9-26; **Event:** year: 1936; month: 7; day: 7; **Record Level:** institutionCode: MZH; basisOfRecord: PreservedSpecimen

#### Diagnosis

Best identified by structures of the male genitalia as illustrated by Collin (1966).

#### Distribution

First recorded from Finland by Zatwarnicki and Kahanpää (2014). Western and Central Europe, also recorded from Bulgaria (Zatwarnicki 2013).

#### Biology

The Finnish specimen was caught on water soldiers (*Stratiotes*), which is a host plant of *H. tarsata* (Przhiboro 2004).

#### Notes

Three most likely conspecific females were caught together with the male.

### Allotrichoma bezzii

Becker, 1896

#### Materials

- **Type status:** Other material. **Occurrence:** catalogNumber: http://id.luomus.fi/GV.16599; recordNumber: 7021; recordedBy: Frey, Richard; individualCount: 1; sex: M; lifeStage: adult; **Taxon:** scientificName: *Allotrichoma bezzii* Becker, 1896; order: Diptera; family: Ephydridae; genus: Allotrichoma; specificEpithet: bezzii; scientificNameAuthorship: Becker, 1896; **Location:** country: Finland; stateProvince: A; municipality: Jomala; locality: Öjen; verbatimLocality: Jomala; decimalLatitude: 60.18; decimalLongitude: 19.99; geodeticDatum: wgs84; coordinateUncertaintyInMeters: 1000; georeferencedBy: Kahanpää, Jere; **Identification:** identifiedBy: Kahanpää, Jere; dateIdentified: 2013-9-26; **Event:** year: 1945; month: 6; day: 19; habitat: shallow sea shore; **Record Level:** institutionCode: MZH; basisOfRecord: PreservedSpecimen
- **Type status:** Other material. **Occurrence:** catalogNumber: http://id.luomus.fi/GV.16598; recordedBy: Frey, Richard; individualCount: 1; sex: M; lifeStage: adult; preparations: gen. prep. on pin; **Taxon:** scientificName: *Allotrichoma bezzii* Becker, 1896; order: Diptera; family: Ephydridae; genus: Allotrichoma; specificEpithet: bezzii; scientificNameAuthorship: Becker, 1896; **Location:** country: Finland; stateProvince: EH; municipality: Ylöjärvi; decimalLatitude: 61.55; decimalLongitude: 23.60; geodeticDatum: wgs84; georeferencedBy: Kahanpää, Jere; **Identification:** identifiedBy: Kahanpää, Jere; dateIdentified: 2013-9-26; **Event:** year: 1922; month: 4; day: 18; **Record Level:** institutionCode: MZH; basisOfRecord: PreservedSpecimen
- **Type status:** Other material. **Occurrence:** catalogNumber: http://id.luomus.fi/GV.16600; recordNumber: 909; recordedBy: Frey, Richard; individualCount: 1; sex: M; lifeStage: adult; **Taxon:** scientificName: *Allotrichoma bezzii* Becker, 1896; order: Diptera; family: Ephydridae; genus: Allotrichoma; specificEpithet: bezzii; scientificNameAuthorship: Becker, 1896; **Location:** country: Russia; stateProvince: Carelian Republic; municipality: Paanajärvi; decimalLatitude: 66.27; decimalLongitude: 29.80; geodeticDatum: wgs84; georeferencedBy: Kahanpää, Jere; **Identification:** identifiedBy: Kahanpää, Jere; dateIdentified: 2013-9-26; **Event:** year: 1939; month: 6; day: 17; **Record Level:** institutionCode: MZH; basisOfRecord: PreservedSpecimen
- **Type status:** Other material. **Occurrence:** catalogNumber: http://id.luomus.fi/GV.16601; recordNumber: 4954; recordedBy: Storå, Ragnar; individualCount: 1; sex: M; lifeStage: adult; **Taxon:** scientificName: *Allotrichoma bezzii* Becker, 1896; order: Diptera; family: Ephydridae; genus: Allotrichoma; specificEpithet: bezzii; scientificNameAuthorship: Becker, 1896; **Location:** country: Finland; stateProvince: KP; municipality: Uusikaarlepyy; verbatimLocality: Nykarleby; decimalLatitude: 63.436; decimalLongitude: 22.675; geodeticDatum: wgs84; georeferencedBy: Kahanpää, Jere; **Identification:** identifiedBy: Kahanpää, Jere; dateIdentified: 2013-9-26; **Event:** year: 1954; month: 5; day: 26; **Record Level:** institutionCode: MZH; basisOfRecord: PreservedSpecimen
- **Type status:** Other material. **Occurrence:** catalogNumber: http://id.luomus.fi/GV.19196; recordNumber: 989; recordedBy: Platonoff, Stephan; individualCount: 1; lifeStage: adult; **Taxon:** scientificName: *Allotrichoma bezzii* Becker, 1896; order: Diptera; family: Ephydridae; genus: Allotrichoma; specificEpithet: bezzii; scientificNameAuthorship: Becker, 1896; **Location:** country: Russia; stateProvince: Republic of Carelia; municipality: Paanajärvi; verbatimLocality: Paanajärvi; decimalLatitude: 66.27; decimalLongitude: 29.80; geodeticDatum: wgs84; georeferencedBy: Kahanpää, Jere; **Identification:** identifiedBy: Krivosheina, M.G.; dateIdentified: 1999; **Record Level:** institutionCode: MZH; basisOfRecord: PreservedSpecimen
- **Type status:** Other material. **Occurrence:** catalogNumber: http://id.luomus.fi/GV.19197; recordNumber: 1697; recordedBy: Frey, Richard; individualCount: 1; lifeStage: adult; **Taxon:** scientificName: *Allotrichoma bezzii* Becker, 1896; order: Diptera; family: Ephydridae; genus: Allotrichoma; specificEpithet: bezzii; scientificNameAuthorship: Becker, 1896; **Location:** country: Finland; stateProvince: V; municipality: Lohja; verbatimLocality: Lojo; decimalLatitude: 60.237; decimalLongitude: 24.007; geodeticDatum: wgs84; georeferencedBy: Kahanpää, Jere; **Identification:** identifiedBy: Krivosheina, M.G.; dateIdentified: 1999; **Record Level:** institutionCode: MZH; basisOfRecord: PreservedSpecimen
- **Type status:** Other material. **Occurrence:** catalogNumber: http://id.luomus.fi/GV.19198; recordNumber: 3118; recordedBy: Frey, Richard; individualCount: 1; lifeStage: adult; **Taxon:** scientificName: *Allotrichoma bezzii* Becker, 1896; order: Diptera; family: Ephydridae; genus: Allotrichoma; specificEpithet: bezzii; scientificNameAuthorship: Becker, 1896; **Location:** country: Finland; stateProvince: V; municipality: Vihti; verbatimLocality: Vichtis; decimalLatitude: 60.424; decimalLongitude: 24.353; geodeticDatum: wgs84; georeferencedBy: Kahanpää, Jere; **Identification:** identifiedBy: Krivosheina, M.G.; dateIdentified: 1999; **Record Level:** institutionCode: MZH; basisOfRecord: PreservedSpecimen
- **Type status:** Other material. **Occurrence:** catalogNumber: http://id.luomus.fi/GV.19199; recordNumber: 1676; recordedBy: Frey, Richard; individualCount: 1; lifeStage: adult; **Taxon:** scientificName: *Allotrichoma bezzii* Becker, 1896; order: Diptera; family: Ephydridae; genus: Allotrichoma; specificEpithet: bezzii; scientificNameAuthorship: Becker, 1896; **Location:** country: Finland; stateProvince: V; municipality: Lohja; verbatimLocality: Lojo; decimalLatitude: 60.237; decimalLongitude: 24.007; geodeticDatum: wgs84; georeferencedBy: Kahanpää, Jere; **Identification:** identifiedBy: Krivosheina, M.G.; dateIdentified: 1999; **Record Level:** institutionCode: MZH; basisOfRecord: PreservedSpecimen
- **Type status:** Other material. **Occurrence:** catalogNumber: http://id.luomus.fi/GV.19200; recordNumber: 1700; recordedBy: Frey, Richard; individualCount: 1; lifeStage: adult; **Taxon:** scientificName: *Allotrichoma bezzii* Becker, 1896; order: Diptera; family: Ephydridae; genus: Allotrichoma; specificEpithet: bezzii; scientificNameAuthorship: Becker, 1896; **Location:** country: Finland; stateProvince: V; municipality: Lohja; verbatimLocality: Lojo; decimalLatitude: 60.237; decimalLongitude: 24.007; geodeticDatum: wgs84; georeferencedBy: Kahanpää, Jere; **Identification:** identifiedBy: Krivosheina, M.G.; dateIdentified: 1999; **Record Level:** institutionCode: MZH; basisOfRecord: PreservedSpecimen
- **Type status:** Other material. **Occurrence:** catalogNumber: http://id.luomus.fi/GV.19201; recordNumber: 2863; recordedBy: Frey, Richard; individualCount: 1; lifeStage: adult; **Taxon:** scientificName: Allotrichoma bezzii Becker, 1896; order: Diptera; family: Ephydridae; genus: Allotrichoma; specificEpithet: bezzii; scientificNameAuthorship: Becker, 1896; **Location:** country: Finland; stateProvince: V; municipality: Lohja; verbatimLocality: Lojo; decimalLatitude: 60.237; decimalLongitude: 24.007; geodeticDatum: wgs84; georeferencedBy: Kahanpää, Jere; **Identification:** identifiedBy: Krivosheina, M.G.; dateIdentified: 1999; **Record Level:** institutionCode: MZH; basisOfRecord: PreservedSpecimen
- **Type status:** Other material. **Occurrence:** catalogNumber: http://id.luomus.fi/GV.19202; recordNumber: 3361; recordedBy: Frey, Richard; individualCount: 1; lifeStage: adult; **Taxon:** scientificName: *Allotrichoma bezzii* Becker, 1896; order: Diptera; family: Ephydridae; genus: Allotrichoma; specificEpithet: bezzii; scientificNameAuthorship: Becker, 1896; **Location:** country: Finland; stateProvince: PPe; municipality: Hailuoto; verbatimLocality: Karlö; decimalLatitude: 65.099; decimalLongitude: 24.750; geodeticDatum: wgs84; georeferencedBy: Kahanpää, Jere; **Identification:** identifiedBy: Krivosheina, M.G.; dateIdentified: 1999; **Record Level:** institutionCode: MZH; basisOfRecord: PreservedSpecimen
- **Type status:** Other material. **Occurrence:** catalogNumber: http://id.luomus.fi/GV.19203; recordNumber: 1887; recordedBy: Storå, Ragnar; individualCount: 1; lifeStage: adult; **Taxon:** scientificName: *Allotrichoma bezzii* Becker, 1896; order: Diptera; family: Ephydridae; genus: Allotrichoma; specificEpithet: bezzii; scientificNameAuthorship: Becker, 1896; **Location:** country: Finland; stateProvince: KP; municipality: Pedersöre; verbatimLocality: Pedersöre; decimalLatitude: 63.531; decimalLongitude: 22.862; geodeticDatum: wgs84; georeferencedBy: Kahanpää, Jere; **Identification:** identifiedBy: Krivosheina, M.G.; dateIdentified: 1999; **Event:** year: 1955; month: 7; day: 22; **Record Level:** institutionCode: MZH; basisOfRecord: PreservedSpecimen
- **Type status:** Other material. **Occurrence:** catalogNumber: http://id.luomus.fi/GV.19204; recordedBy: Tiensuu, Lauri; individualCount: 1; lifeStage: adult; **Taxon:** scientificName: *Allotrichoma bezzii* Becker, 1896; order: Diptera; family: Ephydridae; genus: Allotrichoma; specificEpithet: bezzii; scientificNameAuthorship: Becker, 1896; **Location:** country: Finland; stateProvince: U; municipality: Helsinki; verbatimLocality: U: Helsinki; decimalLatitude: 60.165; decimalLongitude: 24.915; geodeticDatum: wgs84; georeferencedBy: Kahanpää, Jere; **Identification:** identifiedBy: Krivosheina, M.G.; dateIdentified: 1999; **Event:** year: 1949; month: 4; day: 17; **Record Level:** institutionCode: MZH; basisOfRecord: PreservedSpecimen
- **Type status:** Other material. **Occurrence:** catalogNumber: http://id.luomus.fi/GV.19205; recordNumber: 4011; recordedBy: Storå, Ragnar; individualCount: 1; lifeStage: adult; **Taxon:** scientificName: *Allotrichoma bezzii* Becker, 1896; order: Diptera; family: Ephydridae; genus: Allotrichoma; specificEpithet: bezzii; scientificNameAuthorship: Becker, 1896; **Location:** country: Finland; stateProvince: KP; municipality: Pedersöre; verbatimLocality: Pedersöre; decimalLatitude: 63.531; decimalLongitude: 22.862; geodeticDatum: wgs84; georeferencedBy: Kahanpää, Jere; **Identification:** identifiedBy: Krivosheina, M.G.; dateIdentified: 1999; **Event:** year: 1958; month: 6; day: 14; **Record Level:** institutionCode: MZH; basisOfRecord: PreservedSpecimen
- **Type status:** Other material. **Occurrence:** catalogNumber: http://id.luomus.fi/GV.19206; recordedBy: Tiensuu, Lauri; individualCount: 1; lifeStage: adult; **Taxon:** scientificName: *Allotrichoma bezzii* Becker, 1896; order: Diptera; family: Ephydridae; genus: Allotrichoma; specificEpithet: bezzii; scientificNameAuthorship: Becker, 1896; **Location:** country: Finland; stateProvince: U; municipality: Helsinki; verbatimLocality: Helsinki; decimalLatitude: 60.165; decimalLongitude: 24.915; geodeticDatum: wgs84; georeferencedBy: Kahanpää, Jere; **Identification:** identifiedBy: Krivosheina, M.G.; dateIdentified: 1999; **Event:** year: 1949; month: 4; day: 17; **Record Level:** institutionCode: MZH; basisOfRecord: PreservedSpecimen
- **Type status:** Other material. **Occurrence:** catalogNumber: http://id.luomus.fi/GV.19207; recordNumber: 4487; recordedBy: Storå, Ragnar; individualCount: 1; lifeStage: adult; **Taxon:** scientificName: *Allotrichoma bezzii* Becker, 1896; order: Diptera; family: Ephydridae; genus: Allotrichoma; specificEpithet: bezzii; scientificNameAuthorship: Becker, 1896; **Location:** country: Finland; stateProvince: KP; municipality: Pietarsaari; verbatimLocality: Jakobstad; decimalLatitude: 63.704; decimalLongitude: 22.634; geodeticDatum: wgs84; georeferencedBy: Kahanpää, Jere; **Identification:** identifiedBy: Krivosheina, M.G.; dateIdentified: 1999; **Event:** year: 1959; month: 8; day: 12; **Record Level:** institutionCode: MZH; basisOfRecord: PreservedSpecimen
- **Type status:** Other material. **Occurrence:** catalogNumber: http://id.luomus.fi/GV.19208; recordNumber: 2575; recordedBy: Frey, Richard; individualCount: 1; lifeStage: adult; **Taxon:** scientificName: *Allotrichoma bezzii* Becker, 1896; order: Diptera; family: Ephydridae; genus: Allotrichoma; specificEpithet: bezzii; scientificNameAuthorship: Becker, 1896; **Location:** country: Finland; stateProvince: U; municipality: Helsinki; verbatimLocality: H:fors; decimalLatitude: 60.165; decimalLongitude: 24.915; geodeticDatum: wgs84; georeferencedBy: Kahanpää, Jere; **Identification:** identifiedBy: Krivosheina, M.G.; dateIdentified: 1999; **Record Level:** institutionCode: MZH; basisOfRecord: PreservedSpecimen
- **Type status:** Other material. **Occurrence:** catalogNumber: http://id.luomus.fi/GV.19209; recordNumber: 755; recordedBy: Tuomikoski, Risto; individualCount: 1; lifeStage: adult; **Taxon:** scientificName: *Allotrichoma bezzii* Becker, 1896; order: Diptera; family: Ephydridae; genus: Allotrichoma; specificEpithet: bezzii; scientificNameAuthorship: Becker, 1896; **Location:** country: Finland; stateProvince: U; municipality: Helsinki; verbatimLocality: Helsinki; decimalLatitude: 60.165; decimalLongitude: 24.915; geodeticDatum: wgs84; georeferencedBy: Kahanpää, Jere; **Identification:** identifiedBy: Krivosheina, M.G.; dateIdentified: 1999; **Record Level:** institutionCode: MZH; basisOfRecord: PreservedSpecimen
- **Type status:** Other material. **Occurrence:** catalogNumber: http://id.luomus.fi/GV.19210; recordNumber: 41; recordedBy: Tuomikoski, Risto; individualCount: 1; lifeStage: adult; **Taxon:** scientificName: *Allotrichoma bezzii* Becker, 1896; order: Diptera; family: Ephydridae; genus: Allotrichoma; specificEpithet: bezzii; scientificNameAuthorship: Becker, 1896; **Location:** country: Finland; stateProvince: U; municipality: Espoo; verbatimLocality: Espoo; decimalLatitude: 60.251; decimalLongitude: 24.729; geodeticDatum: wgs84; georeferencedBy: Kahanpää, Jere; **Identification:** identifiedBy: Krivosheina, M.G.; dateIdentified: 1999; **Record Level:** institutionCode: MZH; basisOfRecord: PreservedSpecimen
- **Type status:** Other material. **Occurrence:** catalogNumber: http://id.luomus.fi/GV.19211; recordNumber: 4622; recordedBy: Storå, Ragnar; individualCount: 1; lifeStage: adult; **Taxon:** scientificName: *Allotrichoma bezzii* Becker, 1896; order: Diptera; family: Ephydridae; genus: Allotrichoma; specificEpithet: bezzii; scientificNameAuthorship: Becker, 1896; **Location:** country: Finland; stateProvince: KP; municipality: Pedersöre; verbatimLocality: Pedersöre; decimalLatitude: 63.531; decimalLongitude: 22.862; geodeticDatum: wgs84; georeferencedBy: Kahanpää, Jere; **Identification:** identifiedBy: Krivosheina, M.G.; dateIdentified: 1999; **Event:** year: 1954; month: 5; day: 20; **Record Level:** institutionCode: MZH; basisOfRecord: PreservedSpecimen
- **Type status:** Other material. **Occurrence:** catalogNumber: http://id.luomus.fi/GV.19212; recordNumber: 4047; recordedBy: Storå, Ragnar; individualCount: 1; lifeStage: adult; **Taxon:** scientificName: *Allotrichoma bezzii* Becker, 1896; order: Diptera; family: Ephydridae; genus: Allotrichoma; specificEpithet: bezzii; scientificNameAuthorship: Becker, 1896; **Location:** country: Finland; stateProvince: KP; municipality: Pedersöre; verbatimLocality: Pedersöre; decimalLatitude: 63.531; decimalLongitude: 22.862; geodeticDatum: wgs84; georeferencedBy: Kahanpää, Jere; **Identification:** identifiedBy: Krivosheina, M.G.; dateIdentified: 1999; **Event:** year: 1954; month: 5; day: 13; **Record Level:** institutionCode: MZH; basisOfRecord: PreservedSpecimen
- **Type status:** Other material. **Occurrence:** catalogNumber: http://id.luomus.fi/GV.19213; recordNumber: 2826; recordedBy: Frey, Richard; individualCount: 1; lifeStage: adult; **Taxon:** scientificName: *Allotrichoma bezzii* Becker, 1896; order: Diptera; family: Ephydridae; genus: Allotrichoma; specificEpithet: bezzii; scientificNameAuthorship: Becker, 1896; **Location:** country: Finland; stateProvince: V; municipality: Lohja; verbatimLocality: Lojo; decimalLatitude: 60.237; decimalLongitude: 24.007; geodeticDatum: wgs84; georeferencedBy: Kahanpää, Jere; **Identification:** identifiedBy: Krivosheina, M.G.; dateIdentified: 1999; **Record Level:** institutionCode: MZH; basisOfRecord: PreservedSpecimen
- **Type status:** Other material. **Occurrence:** catalogNumber: http://id.luomus.fi/GV.19214; recordNumber: 4660; recordedBy: Storå, Ragnar; individualCount: 1; lifeStage: adult; **Taxon:** scientificName: *Allotrichoma bezzii* Becker, 1896; order: Diptera; family: Ephydridae; genus: Allotrichoma; specificEpithet: bezzii; scientificNameAuthorship: Becker, 1896; **Location:** country: Finland; stateProvince: KP; municipality: Pedersöre; verbatimLocality: Pedersöre; decimalLatitude: 63.531; decimalLongitude: 22.862; geodeticDatum: wgs84; georeferencedBy: Kahanpää, Jere; **Identification:** identifiedBy: Krivosheina, M.G.; dateIdentified: 1999; **Event:** year: 1954; month: 5; day: 20; **Record Level:** institutionCode: MZH; basisOfRecord: PreservedSpecimen
- **Type status:** Other material. **Occurrence:** catalogNumber: http://id.luomus.fi/GV.19215; recordNumber: 4775; recordedBy: Storå, Ragnar; individualCount: 1; lifeStage: adult; **Taxon:** scientificName: *Allotrichoma bezzii* Becker, 1896; order: Diptera; family: Ephydridae; genus: Allotrichoma; specificEpithet: bezzii; scientificNameAuthorship: Becker, 1896; **Location:** country: Finland; stateProvince: KP; municipality: Uusikaarlepyy; verbatimLocality: Nykarleby; decimalLatitude: 63.436; decimalLongitude: 22.675; geodeticDatum: wgs84; georeferencedBy: Kahanpää, Jere; **Identification:** identifiedBy: Krivosheina, M.G.; dateIdentified: 1999; **Record Level:** institutionCode: MZH; basisOfRecord: PreservedSpecimen
- **Type status:** Other material. **Occurrence:** catalogNumber: http://id.luomus.fi/GV.19216; recordNumber: 4764; recordedBy: Storå, Ragnar; individualCount: 1; lifeStage: adult; **Taxon:** scientificName: *Allotrichoma bezzii* Becker, 1896; order: Diptera; family: Ephydridae; genus: Allotrichoma; specificEpithet: bezzii; scientificNameAuthorship: Becker, 1896; **Location:** country: Finland; stateProvince: KP; municipality: Uusikaarlepyy; verbatimLocality: Nykarleby; decimalLatitude: 63.436; decimalLongitude: 22.675; geodeticDatum: wgs84; georeferencedBy: Kahanpää, Jere; **Identification:** identifiedBy: Krivosheina, M.G.; dateIdentified: 1999; **Record Level:** institutionCode: MZH; basisOfRecord: PreservedSpecimen
- **Type status:** Other material. **Occurrence:** catalogNumber: http://id.luomus.fi/GV.19217; recordNumber: 1820; recordedBy: Frey, Richard; individualCount: 1; lifeStage: adult; **Taxon:** scientificName: *Allotrichoma bezzii* Becker, 1896; order: Diptera; family: Ephydridae; genus: Allotrichoma; specificEpithet: bezzii; scientificNameAuthorship: Becker, 1896; **Location:** country: Finland; stateProvince: V; municipality: Sammatti; verbatimLocality: Sammatti; decimalLatitude: 60.323; decimalLongitude: 23.818; geodeticDatum: wgs84; georeferencedBy: Kahanpää, Jere; **Identification:** identifiedBy: Krivosheina, M.G.; dateIdentified: 1999; **Record Level:** institutionCode: MZH; basisOfRecord: PreservedSpecimen
- **Type status:** Other material. **Occurrence:** catalogNumber: http://id.luomus.fi/GV.19218; recordNumber: 386; recordedBy: Frey, Richard; individualCount: 1; lifeStage: adult; **Taxon:** scientificName: *Allotrichoma bezzii* Becker, 1896; order: Diptera; family: Ephydridae; genus: Allotrichoma; specificEpithet: bezzii; scientificNameAuthorship: Becker, 1896; **Location:** country: Finland; stateProvince: Ks; municipality: Kuusamo; verbatimLocality: Oulankaj.; decimalLatitude: 66.261; decimalLongitude: 29.543; geodeticDatum: wgs84; georeferencedBy: Kahanpää, Jere; **Identification:** identifiedBy: Krivosheina, M.G.; dateIdentified: 1999; **Record Level:** institutionCode: MZH; basisOfRecord: PreservedSpecimen
- **Type status:** Other material. **Occurrence:** catalogNumber: http://id.luomus.fi/GV.19219; recordNumber: 1827; recordedBy: Storå, Ragnar; individualCount: 1; lifeStage: adult; **Taxon:** scientificName: *Allotrichoma bezzii* Becker, 1896; order: Diptera; family: Ephydridae; genus: Allotrichoma; specificEpithet: bezzii; scientificNameAuthorship: Becker, 1896; **Location:** country: Finland; stateProvince: KP; municipality: Uusikaarlepyy; verbatimLocality: Nykarleby; decimalLatitude: 63.436; decimalLongitude: 22.675; geodeticDatum: wgs84; georeferencedBy: Kahanpää, Jere; **Identification:** identifiedBy: Krivosheina, M.G.; dateIdentified: 1999; **Event:** year: 1955; month: 7; day: 2; **Record Level:** institutionCode: MZH; basisOfRecord: PreservedSpecimen
- **Type status:** Other material. **Occurrence:** catalogNumber: http://id.luomus.fi/GV.19220; recordNumber: 7590; recordedBy: Storå, Ragnar; individualCount: 1; lifeStage: adult; **Taxon:** scientificName: *Allotrichoma bezzii* Becker, 1896; order: Diptera; family: Ephydridae; genus: Allotrichoma; specificEpithet: bezzii; scientificNameAuthorship: Becker, 1896; **Location:** country: Finland; municipality: Kuortane; verbatimLocality: Kuortane; decimalLatitude: 62.741; decimalLongitude: 23.561; geodeticDatum: wgs84; georeferencedBy: Kahanpää, Jere; **Identification:** identifiedBy: Krivosheina, M.G.; dateIdentified: 1999; **Event:** year: 1955; month: 8; day: 17; **Record Level:** institutionCode: MZH; basisOfRecord: PreservedSpecimen
- **Type status:** Other material. **Occurrence:** catalogNumber: http://id.luomus.fi/GV.19221; recordNumber: 4588; recordedBy: Storå, Ragnar; individualCount: 1; lifeStage: adult; **Taxon:** scientificName: *Allotrichoma bezzii* Becker, 1896; order: Diptera; family: Ephydridae; genus: Allotrichoma; specificEpithet: bezzii; scientificNameAuthorship: Becker, 1896; **Location:** country: Finland; stateProvince: KP; municipality: Pedersöre; verbatimLocality: Pedersöre; decimalLatitude: 63.531; decimalLongitude: 22.862; geodeticDatum: wgs84; georeferencedBy: Kahanpää, Jere; **Identification:** identifiedBy: Krivosheina, M.G.; dateIdentified: 1999; **Event:** year: 1954; month: 5; day: 13; **Record Level:** institutionCode: MZH; basisOfRecord: PreservedSpecimen
- **Type status:** Other material. **Occurrence:** catalogNumber: http://id.luomus.fi/GV.19222; recordedBy: Tiensuu, Lauri; individualCount: 1; lifeStage: adult; **Taxon:** scientificName: *Allotrichoma bezzii* Becker, 1896; order: Diptera; family: Ephydridae; genus: Allotrichoma; specificEpithet: bezzii; scientificNameAuthorship: Becker, 1896; **Location:** country: Finland; stateProvince: U; municipality: Helsinki; verbatimLocality: U: Helsinki; decimalLatitude: 60.165; decimalLongitude: 24.915; geodeticDatum: wgs84; georeferencedBy: Kahanpää, Jere; **Identification:** identifiedBy: Krivosheina, M.G.; dateIdentified: 1999; **Event:** year: 1949; month: 4; day: 17; **Record Level:** institutionCode: MZH; basisOfRecord: PreservedSpecimen
- **Type status:** Other material. **Occurrence:** catalogNumber: http://id.luomus.fi/GV.19223; recordNumber: 888; recordedBy: Frey, Richard; individualCount: 1; lifeStage: adult; **Taxon:** scientificName: *Allotrichoma bezzii* Becker, 1896; order: Diptera; family: Ephydridae; genus: Allotrichoma; specificEpithet: bezzii; scientificNameAuthorship: Becker, 1896; **Location:** country: Russia; stateProvince: Republic of Carelia; municipality: Paanajärvi; verbatimLocality: Paanajärvi; decimalLatitude: 66.27; decimalLongitude: 29.80; geodeticDatum: wgs84; georeferencedBy: Kahanpää, Jere; **Identification:** identifiedBy: Krivosheina, M.G.; dateIdentified: 1999; **Record Level:** institutionCode: MZH; basisOfRecord: PreservedSpecimen

#### Distribution

First recorded from Finland by Zatwarnicki and Kahanpää (2014). Holarctic, widespread in Europe (Zatwarnicki 2013).

#### Notes

All old records of *Allotrichoma laterale* (Loew, 1860) from Finland are misidentifications of *Allotrichoma bezzii* Becker, 1896.

### Ditrichophora calceata

(Meigen, 1830)

#### Materials

- **Type status:** Other material. **Occurrence:** catalogNumber: http://id.luomus.fi/GV.16562; recordedBy: Frey, Richard; individualCount: 1; lifeStage: adult; **Taxon:** scientificName: *Ditrichophora calceata* (Meigen, 1830); order: Diptera; family: Ephydridae; genus: Ditrichophora; specificEpithet: calceata; scientificNameAuthorship: (Meigen, 1830); **Location:** country: Finland; stateProvince: U; municipality: Helsinki; locality: University Botanical gardens; decimalLatitude: 60.17; decimalLongitude: 24.95; geodeticDatum: wgs84; coordinateUncertaintyInMeters: 500; georeferencedBy: Kahanpää, Jere; **Identification:** identifiedBy: Zatwarnicki, Tadeusz; dateIdentified: 09/26/2013; **Event:** eventDate: 1942-7-14; year: 1942; month: 7; day: 14; **Record Level:** institutionCode: MZH; basisOfRecord: PreservedSpecimen
- **Type status:** Other material. **Occurrence:** catalogNumber: http://id.luomus.fi/GV.16561; recordedBy: Tiensuu, Lauri; individualCount: 1; lifeStage: adult; **Taxon:** scientificName: *Ditrichophora calceata* (Meigen, 1830); order: Diptera; family: Ephydridae; genus: Ditrichophora; specificEpithet: calceata; scientificNameAuthorship: (Meigen, 1830); **Location:** country: Russia; stateProvince: Carelian Republic; municipality: Petrosawodsk; verbatimLocality: Äänislinna; decimalLatitude: 61.79; decimalLongitude: 34.35; geodeticDatum: wgs84; georeferencedBy: Kahanpää, Jere; **Identification:** identifiedBy: Kahanpää, Jere; **Event:** eventDate: 1946-8-10; year: 1946; month: 8; day: 10; **Record Level:** institutionCode: MZH; basisOfRecord: PreservedSpecimen

#### Distribution

First recorded from Finland by Zatwarnicki and Kahanpää (2014). A West-Palaearctic species found in almost all European countries and also in North Africa (Zatwarnicki 2013).

#### Notes

Specimen http://id.luomus.fi/GV.16561 is shown in Fig. 1c.

### Hecamedoides unispinosus

(Collin, 1943)

#### Materials

- **Type status:** Other material. **Occurrence:** catalogNumber: http://id.luomus.fi/GV.19147; recordedBy: Frey, Richard; individualCount: 1; lifeStage: adult; **Taxon:** scientificName: *Hecamedoides unispinosus* (Collin, 1943); order: Diptera; family: Ephydridae; genus: Hecamedoides; specificEpithet: unispinosus; scientificNameAuthorship: (Collin, 1943); **Location:** country: Finland; stateProvince: EH; municipality: Kangasala; verbatimLocality: Kangasala; decimalLatitude: 61.496; decimalLongitude: 24.0754; geodeticDatum: wgs84; georeferencedBy: Kahanpää, Jere; **Identification:** identifiedBy: Kahanpää, Jere; **Record Level:** institutionCode: MZH; basisOfRecord: PreservedSpecimen
- **Type status:** Other material. **Occurrence:** catalogNumber: http://id.luomus.fi/GV.19224; recordedBy: Frey, Richard; individualCount: 1; lifeStage: adult; **Taxon:** scientificName: *Hecamedoides unispinosus* (Collin, 1943); order: Diptera; family: Ephydridae; genus: Hecamedoides; specificEpithet: unispinosus; scientificNameAuthorship: (Collin, 1943); **Location:** country: Finland; stateProvince: EH; municipality: Kangasala; verbatimLocality: Kangasala; decimalLatitude: 61.496; decimalLongitude: 24.0754; geodeticDatum: wgs84; georeferencedBy: Kahanpää, Jere; **Identification:** identifiedBy: Zatwarnicki, Th.; **Record Level:** institutionCode: MZH; basisOfRecord: PreservedSpecimen
- **Type status:** Other material. **Occurrence:** catalogNumber: http://id.luomus.fi/GV.19225; recordedBy: Frey, Richard; individualCount: 1; lifeStage: adult; **Taxon:** scientificName: *Hecamedoides unispinosus* (Collin, 1943); order: Diptera; family: Ephydridae; genus: Hecamedoides; specificEpithet: unispinosus; scientificNameAuthorship: (Collin, 1943); **Location:** country: Finland; stateProvince: EH; municipality: Kangasala; verbatimLocality: Kangasala; decimalLatitude: 61.496; decimalLongitude: 24.0754; geodeticDatum: wgs84; georeferencedBy: Kahanpää, Jere; **Identification:** identifiedBy: Zatwarnicki, Th.; **Record Level:** institutionCode: MZH; basisOfRecord: PreservedSpecimen
- **Type status:** Other material. **Occurrence:** catalogNumber: http://id.luomus.fi/GV.19226; recordedBy: Frey, Richard; individualCount: 1; lifeStage: adult; **Taxon:** scientificName: *Hecamedoides unispinosus* (Collin, 1943); order: Diptera; family: Ephydridae; genus: Hecamedoides; specificEpithet: unispinosus; scientificNameAuthorship: (Collin, 1943); **Location:** country: Finland; stateProvince: EH; municipality: Kangasala; verbatimLocality: Kangasala; decimalLatitude: 61.496; decimalLongitude: 24.0754; geodeticDatum: wgs84; georeferencedBy: Kahanpää, Jere; **Identification:** identifiedBy: Zatwarnicki, Th.; **Record Level:** institutionCode: MZH; basisOfRecord: PreservedSpecimen
- **Type status:** Other material. **Occurrence:** catalogNumber: http://id.luomus.fi/GV.19227; recordedBy: Frey, Richard; individualCount: 1; lifeStage: adult; **Taxon:** scientificName: *Hecamedoides unispinosus* (Collin, 1943); order: Diptera; family: Ephydridae; genus: Hecamedoides; specificEpithet: unispinosus; scientificNameAuthorship: (Collin, 1943); **Location:** country: Finland; stateProvince: V; municipality: Lohja; verbatimLocality: Lojo; decimalLatitude: 60.237; decimalLongitude: 24.0078; geodeticDatum: wgs84; georeferencedBy: Kahanpää, Jere; **Identification:** identifiedBy: Zatwarnicki, Th.; **Record Level:** institutionCode: MZH; basisOfRecord: PreservedSpecimen
- **Type status:** Other material. **Occurrence:** catalogNumber: http://id.luomus.fi/GV.19228; recordedBy: Frey, Richard; individualCount: 1; lifeStage: adult; **Taxon:** scientificName: *Hecamedoides unispinosus* (Collin, 1943); order: Diptera; family: Ephydridae; genus: Hecamedoides; specificEpithet: unispinosus; scientificNameAuthorship: (Collin, 1943); **Location:** country: Finland; stateProvince: V; municipality: Lohja; verbatimLocality: Lojo; decimalLatitude: 60.237; decimalLongitude: 24.0078; geodeticDatum: wgs84; georeferencedBy: Kahanpää, Jere; **Identification:** identifiedBy: Zatwarnicki, Th.; **Record Level:** institutionCode: MZH; basisOfRecord: PreservedSpecimen
- **Type status:** Other material. **Occurrence:** catalogNumber: http://id.luomus.fi/GV.19229; recordedBy: Frey, Richard; individualCount: 1; lifeStage: adult; **Taxon:** scientificName: *Hecamedoides unispinosus* (Collin, 1943); order: Diptera; family: Ephydridae; genus: Hecamedoides; specificEpithet: unispinosus; scientificNameAuthorship: (Collin, 1943); **Location:** country: Russia; municipality: Metsäpirtti; verbatimLocality: Metsäpirtti; decimalLatitude: 60.56; decimalLongitude: 30.52; geodeticDatum: wgs84; georeferencedBy: Kahanpää, Jere; **Identification:** identifiedBy: Zatwarnicki, Th.; **Record Level:** institutionCode: MZH; basisOfRecord: PreservedSpecimen
- **Type status:** Other material. **Occurrence:** catalogNumber: http://id.luomus.fi/GV.19230; recordedBy: Frey, Richard; individualCount: 1; lifeStage: adult; **Taxon:** scientificName: *Hecamedoides unispinosus* (Collin, 1943); order: Diptera; family: Ephydridae; genus: Hecamedoides; specificEpithet: unispinosus; scientificNameAuthorship: (Collin, 1943); **Location:** country: Russia; municipality: Metsäpirtti; verbatimLocality: Metsäpirtti; decimalLatitude: 60.56; decimalLongitude: 30.52; geodeticDatum: wgs84; georeferencedBy: Kahanpää, Jere; **Identification:** identifiedBy: Zatwarnicki, Th.; **Record Level:** institutionCode: MZH; basisOfRecord: PreservedSpecimen
- **Type status:** Other material. **Occurrence:** catalogNumber: http://id.luomus.fi/GV.19231; recordedBy: Frey, Richard; individualCount: 1; lifeStage: adult; **Taxon:** scientificName: *Hecamedoides unispinosus* (Collin, 1943); order: Diptera; family: Ephydridae; genus: Hecamedoides; specificEpithet: unispinosus; scientificNameAuthorship: (Collin, 1943); **Location:** country: Russia; municipality: Metsäpirtti; verbatimLocality: Metsäpirtti; decimalLatitude: 60.56; decimalLongitude: 30.52; geodeticDatum: wgs84; georeferencedBy: Kahanpää, Jere; **Identification:** identifiedBy: Zatwarnicki, Th.; **Record Level:** institutionCode: MZH; basisOfRecord: PreservedSpecimen
- **Type status:** Other material. **Occurrence:** catalogNumber: http://id.luomus.fi/GV.19232; recordedBy: Frey, Richard; individualCount: 1; lifeStage: adult; **Taxon:** scientificName: *Hecamedoides unispinosus* (Collin, 1943); order: Diptera; family: Ephydridae; genus: Hecamedoides; specificEpithet: unispinosus; scientificNameAuthorship: (Collin, 1943); **Location:** country: Russia; municipality: Metsäpirtti; verbatimLocality: Metsäpirtti; decimalLatitude: 60.56; decimalLongitude: 30.52; geodeticDatum: wgs84; georeferencedBy: Kahanpää, Jere; **Identification:** identifiedBy: Zatwarnicki, Th.; **Record Level:** institutionCode: MZH; basisOfRecord: PreservedSpecimen
- **Type status:** Other material. **Occurrence:** catalogNumber: http://id.luomus.fi/GV.19233; recordedBy: Frey, Richard; individualCount: 1; lifeStage: adult; **Taxon:** scientificName: *Hecamedoides unispinosus* (Collin, 1943); order: Diptera; family: Ephydridae; genus: Hecamedoides; specificEpithet: unispinosus; scientificNameAuthorship: (Collin, 1943); **Location:** country: Russia; municipality: Metsäpirtti; verbatimLocality: Metsäpirtti; decimalLatitude: 60.56; decimalLongitude: 30.52; geodeticDatum: wgs84; georeferencedBy: Kahanpää, Jere; **Identification:** identifiedBy: Zatwarnicki, Th.; **Record Level:** institutionCode: MZH; basisOfRecord: PreservedSpecimen
- **Type status:** Other material. **Occurrence:** catalogNumber: http://id.luomus.fi/GV.19234; recordedBy: Frey, Richard; individualCount: 1; lifeStage: adult; **Taxon:** scientificName: *Hecamedoides unispinosus* (Collin, 1943); order: Diptera; family: Ephydridae; genus: Hecamedoides; specificEpithet: unispinosus; scientificNameAuthorship: (Collin, 1943); **Location:** country: Russia; municipality: Metsäpirtti; verbatimLocality: Metsäpirtti; decimalLatitude: 60.56; decimalLongitude: 30.52; geodeticDatum: wgs84; georeferencedBy: Kahanpää, Jere; **Identification:** identifiedBy: Zatwarnicki, Th.; **Record Level:** institutionCode: MZH; basisOfRecord: PreservedSpecimen
- **Type status:** Other material. **Occurrence:** catalogNumber: http://id.luomus.fi/GV.19235; recordedBy: Tiensuu, Lauri; individualCount: 1; lifeStage: adult; **Taxon:** scientificName: *Hecamedoides unispinosus* (Collin, 1943); order: Diptera; family: Ephydridae; genus: Hecamedoides; specificEpithet: unispinosus; scientificNameAuthorship: (Collin, 1943); **Location:** country: Russia; municipality: Vaaseni; verbatimLocality: Terijoki; decimalLatitude: 60.96; decimalLongitude: 34.02; geodeticDatum: wgs84; georeferencedBy: Kahanpää, Jere; **Identification:** identifiedBy: Zatwarnicki, Th.; **Event:** year: 1939; month: 6; day: 8; **Record Level:** institutionCode: MZH; basisOfRecord: PreservedSpecimen
- **Type status:** Other material. **Occurrence:** catalogNumber: http://id.luomus.fi/GV.19236; recordedBy: Tiensuu, Lauri; individualCount: 1; lifeStage: adult; **Taxon:** scientificName: *Hecamedoides unispinosus* (Collin, 1943); order: Diptera; family: Ephydridae; genus: Hecamedoides; specificEpithet: unispinosus; scientificNameAuthorship: (Collin, 1943); **Location:** country: Russia; municipality: Vaaseni; verbatimLocality: Vaaseni; decimalLatitude: 60.96; decimalLongitude: 34.02; geodeticDatum: wgs84; georeferencedBy: Kahanpää, Jere; **Identification:** identifiedBy: Zatwarnicki, Th.; **Event:** year: 1942; month: 6; day: 15; **Record Level:** institutionCode: MZH; basisOfRecord: PreservedSpecimen
- **Type status:** Other material. **Occurrence:** catalogNumber: http://id.luomus.fi/GV.19237; recordedBy: Tiensuu, Lauri; individualCount: 1; lifeStage: adult; **Taxon:** scientificName: *Hecamedoides unispinosus* (Collin, 1943); order: Diptera; family: Ephydridae; genus: Hecamedoides; specificEpithet: unispinosus; scientificNameAuthorship: (Collin, 1943); **Location:** country: Russia; municipality: Terijoki; verbatimLocality: Terijoki; decimalLatitude: 60.18; decimalLongitude: 29.7; geodeticDatum: wgs84; georeferencedBy: Kahanpää, Jere; **Identification:** identifiedBy: Zatwarnicki, Th.; **Event:** year: 1939; month: 6; day: 8; **Record Level:** institutionCode: MZH; basisOfRecord: PreservedSpecimen
- **Type status:** Other material. **Occurrence:** catalogNumber: http://id.luomus.fi/GV.19238; recordedBy: Tiensuu, Lauri; individualCount: 1; lifeStage: adult; **Taxon:** scientificName: *Hecamedoides unispinosus* (Collin, 1943); order: Diptera; family: Ephydridae; genus: Hecamedoides; specificEpithet: unispinosus; scientificNameAuthorship: (Collin, 1943); **Location:** country: Russia; municipality: Terijoki; verbatimLocality: Terijoki; decimalLatitude: 60.18; decimalLongitude: 29.7; geodeticDatum: wgs84; georeferencedBy: Kahanpää, Jere; **Identification:** identifiedBy: Zatwarnicki, Th.; **Event:** year: 1939; month: 6; day: 8; **Record Level:** institutionCode: MZH; basisOfRecord: PreservedSpecimen
- **Type status:** Other material. **Occurrence:** catalogNumber: http://id.luomus.fi/GV.19239; recordedBy: Tiensuu, Lauri; individualCount: 1; lifeStage: adult; **Taxon:** scientificName: *Hecamedoides unispinosus* (Collin, 1943); order: Diptera; family: Ephydridae; genus: Hecamedoides; specificEpithet: unispinosus; scientificNameAuthorship: (Collin, 1943); **Location:** country: Russia; municipality: Terijoki; verbatimLocality: Terijoki; decimalLatitude: 60.18; decimalLongitude: 29.7; geodeticDatum: wgs84; georeferencedBy: Kahanpää, Jere; **Identification:** identifiedBy: Zatwarnicki, Th.; **Event:** year: 1939; month: 6; day: 8; **Record Level:** institutionCode: MZH; basisOfRecord: PreservedSpecimen
- **Type status:** Other material. **Occurrence:** catalogNumber: http://id.luomus.fi/GV.19240; recordedBy: Frey, Richard; individualCount: 1; lifeStage: adult; **Taxon:** scientificName: *Hecamedoides unispinosus* (Collin, 1943); order: Diptera; family: Ephydridae; genus: Hecamedoides; specificEpithet: unispinosus; scientificNameAuthorship: (Collin, 1943); **Location:** country: Russia; municipality: Metsäpirtti; verbatimLocality: Metsäpirtti; decimalLatitude: 60.56; decimalLongitude: 30.52; geodeticDatum: wgs84; georeferencedBy: Kahanpää, Jere; **Identification:** identifiedBy: Zatwarnicki, Th.; **Record Level:** institutionCode: MZH; basisOfRecord: PreservedSpecimen
- **Type status:** Other material. **Occurrence:** catalogNumber: http://id.luomus.fi/GV.19241; recordedBy: Tiensuu, Lauri; individualCount: 1; lifeStage: adult; **Taxon:** scientificName: *Hecamedoides unispinosus* (Collin, 1943); order: Diptera; family: Ephydridae; genus: Hecamedoides; specificEpithet: unispinosus; scientificNameAuthorship: (Collin, 1943); **Location:** country: Russia; municipality: Terijoki; verbatimLocality: Terijoki; decimalLatitude: 60.18; decimalLongitude: 29.7; geodeticDatum: wgs84; georeferencedBy: Kahanpää, Jere; **Identification:** identifiedBy: Zatwarnicki, Th.; **Event:** year: 1939; month: 6; day: 8; **Record Level:** institutionCode: MZH; basisOfRecord: PreservedSpecimen
- **Type status:** Other material. **Occurrence:** catalogNumber: http://id.luomus.fi/GV.19242; recordedBy: Tiensuu, Lauri; individualCount: 1; lifeStage: adult; **Taxon:** scientificName: *Hecamedoides unispinosus* (Collin, 1943); order: Diptera; family: Ephydridae; genus: Hecamedoides; specificEpithet: unispinosus; scientificNameAuthorship: (Collin, 1943); **Location:** country: Russia; municipality: Terijoki; verbatimLocality: Terijoki; decimalLatitude: 60.18; decimalLongitude: 29.7; geodeticDatum: wgs84; georeferencedBy: Kahanpää, Jere; **Identification:** identifiedBy: Zatwarnicki, Th.; **Event:** year: 1939; month: 6; day: 8; **Record Level:** institutionCode: MZH; basisOfRecord: PreservedSpecimen
- **Type status:** Other material. **Occurrence:** catalogNumber: http://id.luomus.fi/GV.19243; recordedBy: Tiensuu, Lauri; individualCount: 1; lifeStage: adult; **Taxon:** scientificName: *Hecamedoides unispinosus* (Collin, 1943); order: Diptera; family: Ephydridae; genus: Hecamedoides; specificEpithet: unispinosus; scientificNameAuthorship: (Collin, 1943); **Location:** country: Finland; stateProvince: V; municipality: Lohja; verbatimLocality: Lojo; decimalLatitude: 60.237; decimalLongitude: 24.0078; geodeticDatum: wgs84; georeferencedBy: Kahanpää, Jere; **Identification:** identifiedBy: Zatwarnicki, Th.; **Record Level:** institutionCode: MZH; basisOfRecord: PreservedSpecimen
- **Type status:** Other material. **Occurrence:** catalogNumber: http://id.luomus.fi/GV.19244; recordedBy: Frey, Richard; individualCount: 1; lifeStage: adult; **Taxon:** scientificName: *Hecamedoides unispinosus* (Collin, 1943); order: Diptera; family: Ephydridae; genus: Hecamedoides; specificEpithet: unispinosus; scientificNameAuthorship: (Collin, 1943); **Location:** country: Russia; municipality: Vaaseni; verbatimLocality: Vaaseni; decimalLatitude: 60.96; decimalLongitude: 34.02; geodeticDatum: wgs84; georeferencedBy: Kahanpää, Jere; **Identification:** identifiedBy: Zatwarnicki, Th.; **Event:** year: 1942; month: 6; day: 15; **Record Level:** institutionCode: MZH; basisOfRecord: PreservedSpecimen
- **Type status:** Other material. **Occurrence:** catalogNumber: http://id.luomus.fi/GV.19245; recordedBy: Tiensuu, Lauri; individualCount: 1; lifeStage: adult; **Taxon:** scientificName: *Hecamedoides unispinosus* (Collin, 1943); order: Diptera; family: Ephydridae; genus: Hecamedoides; specificEpithet: unispinosus; scientificNameAuthorship: (Collin, 1943); **Location:** country: Russia; municipality: Terijoki; verbatimLocality: Terijoki; decimalLatitude: 60.18; decimalLongitude: 29.7; geodeticDatum: wgs84; georeferencedBy: Kahanpää, Jere; **Identification:** identifiedBy: Zatwarnicki, Th.; **Event:** year: 1939; month: 6; day: 11; **Record Level:** institutionCode: MZH; basisOfRecord: PreservedSpecimen
- **Type status:** Other material. **Occurrence:** catalogNumber: http://id.luomus.fi/GV.19246; recordedBy: Tiensuu, Lauri; individualCount: 1; lifeStage: adult; **Taxon:** scientificName: *Hecamedoides unispinosus* (Collin, 1943); order: Diptera; family: Ephydridae; genus: Hecamedoides; specificEpithet: unispinosus; scientificNameAuthorship: (Collin, 1943); **Location:** country: Russia; municipality: Vaaseni; verbatimLocality: Vaaseni; decimalLatitude: 60.96; decimalLongitude: 34.02; geodeticDatum: wgs84; georeferencedBy: Kahanpää, Jere; **Identification:** identifiedBy: Zatwarnicki, Th.; **Event:** year: 1942; month: 6; day: 1; **Record Level:** institutionCode: MZH; basisOfRecord: PreservedSpecimen
- **Type status:** Other material. **Occurrence:** catalogNumber: http://id.luomus.fi/GV.19247; recordedBy: Tiensuu, Lauri; individualCount: 1; lifeStage: adult; **Taxon:** scientificName: *Hecamedoides unispinosus* (Collin, 1943); order: Diptera; family: Ephydridae; genus: Hecamedoides; specificEpithet: unispinosus; scientificNameAuthorship: (Collin, 1943); **Location:** country: Finland; stateProvince: V; municipality: Lohja; verbatimLocality: Lojo; decimalLatitude: 60.237; decimalLongitude: 24.0078; geodeticDatum: wgs84; georeferencedBy: Kahanpää, Jere; **Identification:** identifiedBy: Zatwarnicki, Th.; **Record Level:** institutionCode: MZH; basisOfRecord: PreservedSpecimen
- **Type status:** Other material. **Occurrence:** catalogNumber: http://id.luomus.fi/GV.19248; recordedBy: Frey, Richard; individualCount: 1; lifeStage: adult; **Taxon:** scientificName: *Hecamedoides unispinosus* (Collin, 1943); order: Diptera; family: Ephydridae; genus: Hecamedoides; specificEpithet: unispinosus; scientificNameAuthorship: (Collin, 1943); **Location:** country: Finland; stateProvince: V; municipality: Lohja; verbatimLocality: Lojo; decimalLatitude: 60.237; decimalLongitude: 24.0078; geodeticDatum: wgs84; georeferencedBy: Kahanpää, Jere; **Identification:** identifiedBy: Zatwarnicki, Th.; **Record Level:** institutionCode: MZH; basisOfRecord: PreservedSpecimen
- **Type status:** Other material. **Occurrence:** catalogNumber: http://id.luomus.fi/GV.19249; recordedBy: Frey, Richard; individualCount: 1; lifeStage: adult; **Taxon:** scientificName: *Hecamedoides unispinosus* (Collin, 1943); order: Diptera; family: Ephydridae; genus: Hecamedoides; specificEpithet: unispinosus; scientificNameAuthorship: (Collin, 1943); **Location:** country: Finland; stateProvince: V; municipality: Lohja; verbatimLocality: Lojo; decimalLatitude: 60.237; decimalLongitude: 24.0078; geodeticDatum: wgs84; georeferencedBy: Kahanpää, Jere; **Identification:** identifiedBy: Zatwarnicki, Th.; **Record Level:** institutionCode: MZH; basisOfRecord: PreservedSpecimen
- **Type status:** Other material. **Occurrence:** catalogNumber: http://id.luomus.fi/GV.19250; recordedBy: Frey, Richard; individualCount: 1; lifeStage: adult; **Taxon:** scientificName: *Hecamedoides unispinosus* (Collin, 1943); order: Diptera; family: Ephydridae; genus: Hecamedoides; specificEpithet: unispinosus; scientificNameAuthorship: (Collin, 1943); **Location:** country: Finland; stateProvince: V; municipality: Lohja; verbatimLocality: Lojo; decimalLatitude: 60.237; decimalLongitude: 24.0078; geodeticDatum: wgs84; georeferencedBy: Kahanpää, Jere; **Identification:** identifiedBy: Zatwarnicki, Th.; **Record Level:** institutionCode: MZH; basisOfRecord: PreservedSpecimen
- **Type status:** Other material. **Occurrence:** catalogNumber: http://id.luomus.fi/GV.19251; recordedBy: Tiensuu, Lauri; individualCount: 1; lifeStage: adult; **Taxon:** scientificName: *Hecamedoides unispinosus* (Collin, 1943); order: Diptera; family: Ephydridae; genus: Hecamedoides; specificEpithet: unispinosus; scientificNameAuthorship: (Collin, 1943); **Location:** country: Finland; stateProvince: U; municipality: Helsinki; locality: Botanical gardens; verbatimLocality: Helsinki, Hortus Bot.; decimalLatitude: 60.174; decimalLongitude: 24.9508; geodeticDatum: wgs84; georeferencedBy: Kahanpää, Jere; **Identification:** identifiedBy: Zatwarnicki, Th.; **Event:** year: 1939; month: 6; day: 5; **Record Level:** institutionCode: MZH; basisOfRecord: PreservedSpecimen
- **Type status:** Other material. **Occurrence:** catalogNumber: http://id.luomus.fi/GV.19252; recordedBy: Storå, Ragnar; individualCount: 1; lifeStage: adult; **Taxon:** scientificName: *Hecamedoides unispinosus* (Collin, 1943); order: Diptera; family: Ephydridae; genus: Hecamedoides; specificEpithet: unispinosus; scientificNameAuthorship: (Collin, 1943); **Location:** country: Finland; stateProvince: KP; municipality: Pedersöre; verbatimLocality: Pedersöre; decimalLatitude: 63.531; decimalLongitude: 22.8625; geodeticDatum: wgs84; georeferencedBy: Kahanpää, Jere; **Identification:** identifiedBy: Zatwarnicki, Th.; **Event:** year: 1958; month: 6; day: 14; **Record Level:** institutionCode: MZH; basisOfRecord: PreservedSpecimen
- **Type status:** Other material. **Occurrence:** catalogNumber: http://id.luomus.fi/GV.19253; recordedBy: Storå, Ragnar; individualCount: 1; lifeStage: adult; **Taxon:** scientificName: *Hecamedoides unispinosus* (Collin, 1943); order: Diptera; family: Ephydridae; genus: Hecamedoides; specificEpithet: unispinosus; scientificNameAuthorship: (Collin, 1943); **Location:** country: Finland; stateProvince: KP; municipality: Pedersöre; verbatimLocality: Pedersöre; decimalLatitude: 63.531; decimalLongitude: 22.8625; geodeticDatum: wgs84; georeferencedBy: Kahanpää, Jere; **Identification:** identifiedBy: Zatwarnicki, Th.; **Event:** year: 1952; month: 6; day: 28; **Record Level:** institutionCode: MZH; basisOfRecord: PreservedSpecimen
- **Type status:** Other material. **Occurrence:** catalogNumber: http://id.luomus.fi/GV.19254; recordedBy: Storå, Ragnar; individualCount: 1; lifeStage: adult; **Taxon:** scientificName: *Hecamedoides unispinosus* (Collin, 1943); order: Diptera; family: Ephydridae; genus: Hecamedoides; specificEpithet: unispinosus; scientificNameAuthorship: (Collin, 1943); **Location:** country: Finland; stateProvince: KP; municipality: Pedersöre; verbatimLocality: Pedersöre; decimalLatitude: 63.531; decimalLongitude: 22.8625; geodeticDatum: wgs84; georeferencedBy: Kahanpää, Jere; **Identification:** identifiedBy: Zatwarnicki, Th.; **Event:** year: 1952; month: 6; day: 28; **Record Level:** institutionCode: MZH; basisOfRecord: PreservedSpecimen
- **Type status:** Other material. **Occurrence:** catalogNumber: http://id.luomus.fi/GV.19255; recordedBy: Storå, Ragnar; individualCount: 1; lifeStage: adult; **Taxon:** scientificName: *Hecamedoides unispinosus* (Collin, 1943); order: Diptera; family: Ephydridae; genus: Hecamedoides; specificEpithet: unispinosus; scientificNameAuthorship: (Collin, 1943); **Location:** country: Finland; stateProvince: KP; municipality: Pedersöre; verbatimLocality: Pedersöre; decimalLatitude: 63.531; decimalLongitude: 22.8625; geodeticDatum: wgs84; georeferencedBy: Kahanpää, Jere; **Identification:** identifiedBy: Zatwarnicki, Th.; **Event:** year: 1952; month: 6; day: 28; **Record Level:** institutionCode: MZH; basisOfRecord: PreservedSpecimen
- **Type status:** Other material. **Occurrence:** catalogNumber: http://id.luomus.fi/GV.19256; recordedBy: Storå, Ragnar; individualCount: 1; lifeStage: adult; **Taxon:** scientificName: *Hecamedoides unispinosus* (Collin, 1943); order: Diptera; family: Ephydridae; genus: Hecamedoides; specificEpithet: unispinosus; scientificNameAuthorship: (Collin, 1943); **Location:** country: Finland; stateProvince: KP; municipality: Pedersöre; verbatimLocality: Pedersöre; decimalLatitude: 63.531; decimalLongitude: 22.8625; geodeticDatum: wgs84; georeferencedBy: Kahanpää, Jere; **Identification:** identifiedBy: Zatwarnicki, Th.; **Event:** year: 1952; month: 6; day: 28; **Record Level:** institutionCode: MZH; basisOfRecord: PreservedSpecimen
- **Type status:** Other material. **Occurrence:** catalogNumber: http://id.luomus.fi/GV.19257; recordedBy: Frey, Richard; individualCount: 1; lifeStage: adult; **Taxon:** scientificName: *Hecamedoides unispinosus* (Collin, 1943); order: Diptera; family: Ephydridae; genus: Hecamedoides; specificEpithet: unispinosus; scientificNameAuthorship: (Collin, 1943); **Location:** country: Finland; stateProvince: V; municipality: Vihti; verbatimLocality: Vichtis; decimalLatitude: 60.424; decimalLongitude: 24.3539; geodeticDatum: wgs84; georeferencedBy: Kahanpää, Jere; **Identification:** identifiedBy: Zatwarnicki, Th.; **Record Level:** institutionCode: MZH; basisOfRecord: PreservedSpecimen
- **Type status:** Other material. **Occurrence:** catalogNumber: http://id.luomus.fi/GV.19258; recordedBy: Frey, Richard; individualCount: 1; lifeStage: adult; **Taxon:** scientificName: *Hecamedoides unispinosus* (Collin, 1943); order: Diptera; family: Ephydridae; genus: Hecamedoides; specificEpithet: unispinosus; scientificNameAuthorship: (Collin, 1943); **Location:** country: Finland; stateProvince: EH; municipality: Kangasala; verbatimLocality: Kangasala; decimalLatitude: 61.496; decimalLongitude: 24.0754; geodeticDatum: wgs84; georeferencedBy: Kahanpää, Jere; **Identification:** identifiedBy: Zatwarnicki, Th.; **Record Level:** institutionCode: MZH; basisOfRecord: PreservedSpecimen
- **Type status:** Other material. **Occurrence:** catalogNumber: http://id.luomus.fi/GV.19259; recordedBy: Frey, Richard; individualCount: 1; lifeStage: adult; **Taxon:** scientificName: *Hecamedoides unispinosus* (Collin, 1943); order: Diptera; family: Ephydridae; genus: Hecamedoides; specificEpithet: unispinosus; scientificNameAuthorship: (Collin, 1943); **Location:** country: Finland; stateProvince: EH; municipality: Kangasala; verbatimLocality: Kangasala; decimalLatitude: 61.496; decimalLongitude: 24.0754; geodeticDatum: wgs84; georeferencedBy: Kahanpää, Jere; **Identification:** identifiedBy: Zatwarnicki, Th.; **Record Level:** institutionCode: MZH; basisOfRecord: PreservedSpecimen

#### Diagnosis

Two of the North European *Hecamedoides* species can be identified by the number of short, blunt anteroventral spine-like setae on the fore femur: one in *H. unispinosus*, several in *H. glaucellus* (Stenhammar, 1844). *Hecamedoides kelmorum* Stuke, 2011, a species recently described from Germany, also has a black antenna and a single anteroventral spine (Stuke 2011). *Hecamedoides unispinosus* and *H. kelmorum* are best identified by structures of the male genitalia. Two Finnish males (http://id.luomus.fi/GV.19243 and http://id.luomus.fi/GV.19235) were dissected to confirm their identification. The gonite of these specimens is clearly of the *H. unispinosus* type.

#### Distribution

A Holarctic species. Recorded from Finland by Kahanpää 2013. New for Russia.

#### Taxon discussion

*Hecamedoides unispinosus* is much more common in Finland than *H. glaucellus*. The Fennica collection of MZH has only three specimens of the latter species: http://id.luomus.fi/GV.19146 from Turku (see Fig. 2b) and two specimens from Terijoki (=Russia, Leningrad Oblast, Zelenogorsk).

### Pelina norvegica

Dahl, 1975

#### Materials

- **Type status:** Other material. **Occurrence:** catalogNumber: http://id.luomus.fi/GV.19162; recordedBy: Frey, Richard; individualCount: 1; sex: M; lifeStage: adult; **Taxon:** scientificName: *Pelina norvegica* Dahl, 1975; order: Diptera; family: Ephydridae; genus: Pelina; specificEpithet: norvegica; scientificNameAuthorship: Dahl, 1975; **Location:** country: Russia; stateProvince: Murmansk Oblast; locality: Yläluostari; verbatimLocality: Petsamo; decimalLatitude: 69.4; decimalLongitude: 31.11; geodeticDatum: wgs84; georeferencedBy: Kahanpää, Jere; **Identification:** identifiedBy: Kahanpää, Jere; dateIdentified: 09/26/2013; **Event:** year: 1930; month: 7; day: 12; habitat: riverside; **Record Level:** institutionCode: MZH; basisOfRecord: PreservedSpecimen
- **Type status:** Other material. **Occurrence:** catalogNumber: http://id.luomus.fi/GV.19163; recordedBy: Frey, Richard; individualCount: 1; sex: M; lifeStage: adult; **Taxon:** scientificName: *Pelina norvegica* Dahl, 1975; order: Diptera; family: Ephydridae; genus: Pelina; specificEpithet: norvegica; scientificNameAuthorship: Dahl, 1975; **Location:** country: Russia; stateProvince: Murmansk Oblast; locality: Yläluostari; verbatimLocality: Petsamo; decimalLatitude: 69.4; decimalLongitude: 31.11; geodeticDatum: wgs84; georeferencedBy: Kahanpää, Jere; **Identification:** identifiedBy: Kahanpää, Jere; dateIdentified: 09/26/2013; **Event:** year: 1930; month: 7; day: 12; habitat: riverside; **Record Level:** institutionCode: MZH; basisOfRecord: PreservedSpecimen

#### Distribution

Previously recorded from Norway, Germany and Slovakia (Zatwarnicki 2013). New for Russia.

#### Notes

Specimen http://id.luomus.fi/GV.19162 of *Pelina norvegica* is shown in Fig. 1d.

### Parydra (Chaetoapnea) mitis

(Cresson, 1930)

#### Materials

- **Type status:** Other material. **Occurrence:** catalogNumber: http://id.luomus.fi/GV.16564; recordedBy: Albrecht, Anders; individualCount: 1; lifeStage: adult; **Taxon:** scientificName: *Parydra mitis* (Cresson, 1930); order: Diptera; family: Ephydridae; genus: Parydra; specificEpithet: mitis; scientificNameAuthorship: (Cresson, 1930); **Location:** country: Finland; stateProvince: U; municipality: Sipoo; locality: Sjöskog; verbatimCoordinates: 6690:3385; geodeticDatum: wgs84; georeferencedBy: Kahanpää, Jere; **Identification:** identifiedBy: Zatwarnicki, T.; **Event:** year: 1975; month: 5; day: 6; **Record Level:** institutionCode: MZH; basisOfRecord: PreservedSpecimen
- **Type status:** Other material. **Occurrence:** catalogNumber: http://id.luomus.fi/GV.16565; recordedBy: Tiensuu, Lauri; individualCount: 1; lifeStage: adult; **Taxon:** scientificName: *Parydra mitis* (Cresson, 1930); order: Diptera; family: Ephydridae; genus: Parydra; specificEpithet: mitis; scientificNameAuthorship: (Cresson, 1930); **Location:** country: Russia; municipality: Vaaseni; geodeticDatum: wgs84; georeferencedBy: Kahanpää, Jere; **Identification:** identifiedBy: Zatwarnicki, T.; **Event:** year: 1942; month: 5; day: 2; **Record Level:** institutionCode: MZH; basisOfRecord: PreservedSpecimen
- **Type status:** Other material. **Occurrence:** catalogNumber: http://id.luomus.fi/GV.16566; recordedBy: Tiensuu, Lauri; individualCount: 1; lifeStage: adult; **Taxon:** scientificName: *Parydra mitis* (Cresson, 1930); order: Diptera; family: Ephydridae; genus: Parydra; specificEpithet: mitis; scientificNameAuthorship: (Cresson, 1930); **Location:** country: Finland; stateProvince: U; municipality: Helsinki; locality: Herttoniemi; geodeticDatum: wgs84; georeferencedBy: Kahanpää, Jere; **Identification:** identifiedBy: Zatwarnicki, T.; **Event:** year: 1945; month: 5; day: 16; **Record Level:** institutionCode: MZH; basisOfRecord: PreservedSpecimen
- **Type status:** Other material. **Occurrence:** catalogNumber: http://id.luomus.fi/GV.16567; recordedBy: Tiensuu, Lauri; individualCount: 1; lifeStage: adult; **Taxon:** scientificName: *Parydra mitis* (Cresson, 1930); order: Diptera; family: Ephydridae; genus: Parydra; specificEpithet: mitis; scientificNameAuthorship: (Cresson, 1930); **Location:** country: Finland; stateProvince: EK; municipality: Vehkalahti; geodeticDatum: wgs84; georeferencedBy: Kahanpää, Jere; **Identification:** identifiedBy: Zatwarnicki, T.; **Event:** year: 1945; month: 4; day: 9; **Record Level:** institutionCode: MZH; basisOfRecord: PreservedSpecimen
- **Type status:** Other material. **Occurrence:** catalogNumber: http://id.luomus.fi/GV.16568; recordedBy: Tiensuu, Lauri; individualCount: 1; lifeStage: adult; **Taxon:** scientificName: *Parydra mitis* (Cresson, 1930); order: Diptera; family: Ephydridae; genus: Parydra; specificEpithet: mitis; scientificNameAuthorship: (Cresson, 1930); **Location:** country: Finland; stateProvince: U; municipality: Helsinki; locality: Herttoniemi; geodeticDatum: wgs84; georeferencedBy: Kahanpää, Jere; **Identification:** identifiedBy: Zatwarnicki, T.; **Event:** year: 1945; month: 5; day: 16; **Record Level:** institutionCode: MZH; basisOfRecord: PreservedSpecimen
- **Type status:** Other material. **Occurrence:** catalogNumber: http://id.luomus.fi/GV.16569; recordedBy: Lundström, Carl; individualCount: 1; lifeStage: adult; **Taxon:** scientificName: *Parydra mitis* (Cresson, 1930); order: Diptera; family: Ephydridae; genus: Parydra; specificEpithet: mitis; scientificNameAuthorship: (Cresson, 1930); **Location:** country: Finland; stateProvince: PS; municipality: Maaninka; locality: Tuovilanlahti; geodeticDatum: wgs84; georeferencedBy: Kahanpää, Jere; **Identification:** identifiedBy: Zatwarnicki, T.; **Event:** year: 1865; **Record Level:** institutionCode: MZH; basisOfRecord: PreservedSpecimen
- **Type status:** Other material. **Occurrence:** catalogNumber: http://id.luomus.fi/GV.16570; recordedBy: Frey, Richard; individualCount: 1; lifeStage: adult; **Taxon:** scientificName: *Parydra mitis* (Cresson, 1930); order: Diptera; family: Ephydridae; genus: Parydra; specificEpithet: mitis; scientificNameAuthorship: (Cresson, 1930); **Location:** country: Russia; stateProvince: Republic of Carelia; municipality: Paanajärvi; locality: behind Takalo farm; decimalLatitude: 66.26; decimalLongitude: 29.78; geodeticDatum: wgs84; georeferencedBy: Kahanpää, Jere; **Identification:** identifiedBy: Zatwarnicki, T.; **Event:** year: 1939; month: 17; day: 6; habitat: lake shore; **Record Level:** institutionCode: MZH; basisOfRecord: PreservedSpecimen
- **Type status:** Other material. **Occurrence:** catalogNumber: http://id.luomus.fi/GV.16571; recordedBy: Storå, Ragnar; individualCount: 1; lifeStage: adult; **Taxon:** scientificName: *Parydra mitis* (Cresson, 1930); order: Diptera; family: Ephydridae; genus: Parydra; specificEpithet: mitis; scientificNameAuthorship: (Cresson, 1930); **Location:** country: Finland; stateProvince: KP; municipality: Uusikaarlepyy; geodeticDatum: wgs84; georeferencedBy: Kahanpää, Jere; **Identification:** identifiedBy: Zatwarnicki, T.; **Event:** year: 1954; **Record Level:** institutionCode: MZH; basisOfRecord: PreservedSpecimen
- **Type status:** Other material. **Occurrence:** catalogNumber: http://id.luomus.fi/GV.16572; recordedBy: Frey, Richard; individualCount: 1; lifeStage: adult; **Taxon:** scientificName: *Parydra mitis* (Cresson, 1930); order: Diptera; family: Ephydridae; genus: Parydra; specificEpithet: mitis; scientificNameAuthorship: (Cresson, 1930); **Location:** country: Russia; stateProvince: Republic of Carelia; municipality: Paanajärvi; locality: hotel; geodeticDatum: wgs84; georeferencedBy: Kahanpää, Jere; **Identification:** identifiedBy: Zatwarnicki, T.; **Event:** year: 1939; month: 7; day: 6; **Record Level:** institutionCode: MZH; basisOfRecord: PreservedSpecimen
- **Type status:** Other material. **Occurrence:** catalogNumber: http://id.luomus.fi/GV.16573; recordedBy: Storå, Ragnar; individualCount: 1; lifeStage: adult; **Taxon:** scientificName: *Parydra mitis* (Cresson, 1930); order: Diptera; family: Ephydridae; genus: Parydra; specificEpithet: mitis; scientificNameAuthorship: (Cresson, 1930); **Location:** country: Finland; stateProvince: KP; municipality: Uusikaarlepyy; geodeticDatum: wgs84; georeferencedBy: Kahanpää, Jere; **Identification:** identifiedBy: Zatwarnicki, T.; **Event:** year: 1954-1955; **Record Level:** institutionCode: MZH; basisOfRecord: PreservedSpecimen
- **Type status:** Other material. **Occurrence:** catalogNumber: http://id.luomus.fi/GV.16574; recordedBy: Frey, Richard; individualCount: 1; lifeStage: adult; **Taxon:** scientificName: *Parydra mitis* (Cresson, 1930); order: Diptera; family: Ephydridae; genus: Parydra; specificEpithet: mitis; scientificNameAuthorship: (Cresson, 1930); **Location:** country: Finland; stateProvince: U; municipality: Helsinki; locality: Munkkiniemi; geodeticDatum: wgs84; georeferencedBy: Kahanpää, Jere; **Identification:** identifiedBy: Zatwarnicki, T.; **Event:** year: 1941; month: 5; day: 4; habitat: a herb-rich forest with a stream; **Record Level:** institutionCode: MZH; basisOfRecord: PreservedSpecimen

#### Distribution

First recorded from Finland by Zatwarnicki and Kahanpää (2014). Widespread in Europe, it is also found in North Africa (Zatwarnicki 2013). The illustrated record by Kahanpää (2013) represents *Parydra nigritarsis* Strobl, 1893.

#### Notes

Fig. 3c illustrates specimen http://id.luomus.fi/GV.16525.

### Calocoenia paurosoma

(Sturtevant & Wheeler, 1954)

#### Materials

- **Type status:** Other material. **Occurrence:** catalogNumber: http://id.luomus.fi/GV.18970; recordNumber: 6643; recordedBy: Storå, Ragnar; individualCount: 1; sex: M; lifeStage: adult; **Taxon:** scientificName: *Calocoenia paurosoma* (Sturdevant & Wheeler, 1954); order: Diptera; family: Ephydridae; genus: Calocoenia; specificEpithet: paurosoma; scientificNameAuthorship: (Sturdevant & Wheeler, 1954); **Location:** country: Finland; stateProvince: EnL; municipality: Enontekiö; locality: Kilpisjärvi; decimalLatitude: 69.0; decimalLongitude: 20.9; geodeticDatum: wgs84; coordinateUncertaintyInMeters: 4000; georeferencedBy: Kahanpää, Jere; **Identification:** identifiedBy: Krivosheina, Marina G.; dateIdentified: 2004; **Event:** year: 1954; month: 8; day: 2; **Record Level:** institutionCode: MZH; basisOfRecord: PreservedSpecimen
- **Type status:** Other material. **Occurrence:** catalogNumber: http://id.luomus.fi/GV.16463; recordNumber: 1106; recordedBy: Frey, Richard; individualCount: 1; lifeStage: adult; **Taxon:** scientificName: *Calocoenia paurosoma* (Sturdevant & Wheeler, 1954); order: Diptera; family: Ephydridae; genus: Calocoenia; specificEpithet: paurosoma; scientificNameAuthorship: (Sturdevant & Wheeler, 1954); **Location:** country: Finland; stateProvince: EnL; municipality: Enontekiö; locality: Kilpisjärvi, Malla; decimalLatitude: 69.09; decimalLongitude: 20.70; geodeticDatum: wgs84; coordinateUncertaintyInMeters: 4000; georeferencedBy: Kahanpää, Jere; **Identification:** identifiedBy: Kahanpää, Jere; dateIdentified: 09/26/2013; **Event:** year: 1929; month: 7; day: 15; habitat: regio subalpina; **Record Level:** institutionCode: MZH; basisOfRecord: PreservedSpecimen
- **Type status:** Other material. **Occurrence:** catalogNumber: http://id.luomus.fi/GV.16457; recordNumber: 392; recordedBy: Frey, Richard; individualCount: 1; lifeStage: adult; **Taxon:** scientificName: *Calocoenia paurosoma* (Sturdevant & Wheeler, 1954); order: Diptera; family: Ephydridae; genus: Calocoenia; specificEpithet: paurosoma; scientificNameAuthorship: (Sturdevant & Wheeler, 1954); **Location:** country: Finland; stateProvince: Ks; municipality: Kuusamo; locality: Kuusamo town, Niilo-oja; locationRemarks: N side of Niilo-oja; decimalLatitude: 65.97; decimalLongitude: 29.18; geodeticDatum: wgs84; coordinateUncertaintyInMeters: 500; georeferencedBy: Kahanpää, Jere; **Identification:** identifiedBy: Kahanpää, Jere; dateIdentified: 09/26/2013; **Event:** year: 1917; month: 6; day: 9; **Record Level:** institutionCode: MZH; basisOfRecord: PreservedSpecimen
- **Type status:** Other material. **Occurrence:** catalogNumber: http://id.luomus.fi/GV.16459; recordNumber: 1115; recordedBy: Frey, Richard; individualCount: 1; lifeStage: adult; **Taxon:** scientificName: *Calocoenia paurosoma* (Sturdevant & Wheeler, 1954); order: Diptera; family: Ephydridae; genus: Calocoenia; specificEpithet: paurosoma; scientificNameAuthorship: (Sturdevant & Wheeler, 1954); **Location:** country: Finland; stateProvince: KemLl; municipality: Kittilä; locality: Alakyrö; decimalLatitude: 68.07; decimalLongitude: 24.39; geodeticDatum: wgs84; georeferencedBy: Kahanpää, Jere; **Identification:** identifiedBy: Kahanpää, Jere; dateIdentified: 09/26/2013; **Event:** year: 1911; month: 6; day: 19; **Record Level:** institutionCode: MZH; basisOfRecord: PreservedSpecimen
- **Type status:** Other material. **Occurrence:** catalogNumber: http://id.luomus.fi/GV.16456; recordNumber: 1153; recordedBy: Lundström, Carl; individualCount: 1; lifeStage: adult; **Taxon:** scientificName: *Calocoenia paurosoma* (Sturdevant & Wheeler, 1954); order: Diptera; family: Ephydridae; genus: Calocoenia; specificEpithet: paurosoma; scientificNameAuthorship: (Sturdevant & Wheeler, 1954); **Location:** country: Finland; stateProvince: PS; municipality: Nilsiä; decimalLatitude: 63.2; decimalLongitude: 28.1; geodeticDatum: wgs84; georeferencedBy: Kahanpää, Jere; **Identification:** identifiedBy: Kahanpää, Jere; dateIdentified: 09/26/2013; **Event:** year: 1865; month: 6; day: 27; **Record Level:** institutionCode: MZH; basisOfRecord: PreservedSpecimen
- **Type status:** Other material. **Occurrence:** catalogNumber: http://id.luomus.fi/GV.16462; recordNumber: 712; recordedBy: Frey, Richard; individualCount: 1; lifeStage: adult; **Taxon:** scientificName: *Calocoenia paurosoma* (Sturdevant & Wheeler, 1954); order: Diptera; family: Ephydridae; genus: Calocoenia; specificEpithet: paurosoma; scientificNameAuthorship: (Sturdevant & Wheeler, 1954); **Location:** country: Russia; stateProvince: Carelian Republic; municipality: Paanajärvi; locality: bog behind Takalo farm; decimalLatitude: 66.26; decimalLongitude: 29.78; geodeticDatum: wgs84; coordinateUncertaintyInMeters: 2000; georeferencedBy: Kahanpää, Jere; **Identification:** identifiedBy: Kahanpää, Jere; dateIdentified: 09/26/2013; **Event:** year: 1937; month: 6; day: 24; habitat: a bog; **Record Level:** institutionCode: MZH; basisOfRecord: PreservedSpecimen
- **Type status:** Other material. **Occurrence:** catalogNumber: http://id.luomus.fi/GV.16458; recordNumber: 673; recordedBy: Frey, Richard; individualCount: 1; lifeStage: adult; **Taxon:** scientificName: *Calocoenia paurosoma* (Sturdevant & Wheeler, 1954); order: Diptera; family: Ephydridae; genus: Calocoenia; specificEpithet: paurosoma; scientificNameAuthorship: (Sturdevant & Wheeler, 1954); **Location:** country: Russia; stateProvince: Carelian Republic; municipality: Paanajärvi; locality: bog behind Takalo farm; decimalLatitude: 66.26; decimalLongitude: 29.78; geodeticDatum: wgs84; coordinateUncertaintyInMeters: 2000; georeferencedBy: Kahanpää, Jere; **Identification:** identifiedBy: Kahanpää, Jere; dateIdentified: 09/26/2013; **Event:** year: 1937; month: 6; day: 24; habitat: a bog; **Record Level:** institutionCode: MZH; basisOfRecord: PreservedSpecimen
- **Type status:** Other material. **Occurrence:** catalogNumber: http://id.luomus.fi/GV.16460; recordNumber: 2492; recordedBy: Frey, Richard; individualCount: 1; lifeStage: adult; **Taxon:** scientificName: *Calocoenia paurosoma* (Sturdevant & Wheeler, 1954); order: Diptera; family: Ephydridae; genus: Calocoenia; specificEpithet: paurosoma; scientificNameAuthorship: (Sturdevant & Wheeler, 1954); **Location:** country: Russia; stateProvince: Murmansk Oblast; municipality: Kantalahti; decimalLatitude: 67.2; decimalLongitude: 32.4; geodeticDatum: wgs84; georeferencedBy: Kahanpää, Jere; **Identification:** identifiedBy: Kahanpää, Jere; dateIdentified: 09/26/2013; **Event:** year: 1913; month: 6; day: 28; **Record Level:** institutionCode: MZH; basisOfRecord: PreservedSpecimen
- **Type status:** Other material. **Occurrence:** catalogNumber: http://id.luomus.fi/GV.16461; recordNumber: 961; recordedBy: Hellén, Wolter; individualCount: 1; lifeStage: adult; **Taxon:** scientificName: *Calocoenia paurosoma* (Sturdevant & Wheeler, 1954); order: Diptera; family: Ephydridae; genus: Calocoenia; specificEpithet: paurosoma; scientificNameAuthorship: (Sturdevant & Wheeler, 1954); **Location:** country: Russia; stateProvince: Murmansk Oblast; municipality: Kantalahti; decimalLatitude: 67.2; decimalLongitude: 32.4; geodeticDatum: wgs84; georeferencedBy: Kahanpää, Jere; **Identification:** identifiedBy: Kahanpää, Jere; dateIdentified: 09/26/2013; **Event:** year: 1913; month: 6; day: 27; **Record Level:** institutionCode: MZH; basisOfRecord: PreservedSpecimen

#### Diagnosis

At first glance this species resembles the genus *Coenia*, but it has five dorsocentral setae. Specimen http://id.luomus.fi/GV.16455 is illustrated in Fig. 4b, d.

#### Distribution

New for Russia. North Holarctic, known in Europe from Norway, Sweden and Finland (Zatwarnicki 2013). First noted from Finland by Krivosheina (2000) from Kilpisjärvi. The new records presented here expand the known European distribution of the species both south- and eastwards.

### Coenia vulgata

Krivosheina, 2001

#### Materials

- **Type status:** Other material. **Occurrence:** catalogNumber: http://id.luomus.fi/GV.16464; recordNumber: 174; recordedBy: Wuorentaus, Yrjö; individualCount: 1; sex: M; lifeStage: adult; preparations: gen. prep. on pin; **Taxon:** scientificName: *Coenia vulgata* Krivosheina, 2001; order: Diptera; family: Ephydridae; genus: Coenia; specificEpithet: vulgata; scientificNameAuthorship: Krivosheina, 2001; **Location:** country: Finland; stateProvince: PPe; municipality: Oulu; decimalLatitude: 65.01; decimalLongitude: 25.41; geodeticDatum: wgs84; coordinateUncertaintyInMeters: 10000; georeferencedBy: Kahanpää, Jere; **Identification:** identifiedBy: Kahanpää, Jere; dateIdentified: 09/26/2013; **Event:** year: 1941; month: 4; day: 27; **Record Level:** institutionCode: MZH; basisOfRecord: PreservedSpecimen
- **Type status:** Other material. **Occurrence:** catalogNumber: http://id.luomus.fi/GV.16465; recordNumber: 775; recordedBy: Frey, Richard; individualCount: 1; sex: M; lifeStage: adult; **Taxon:** scientificName: *Coenia vulgata* Krivosheina, 2001; order: Diptera; family: Ephydridae; genus: Coenia; specificEpithet: vulgata; scientificNameAuthorship: Krivosheina, 2001; **Location:** country: Finland; stateProvince: PPe; municipality: Hailuoto; locality: Kirkkolahti; decimalLatitude: 64.98; decimalLongitude: 24.72; geodeticDatum: wgs84; coordinateUncertaintyInMeters: 2000; georeferencedBy: Kahanpää, Jere; **Identification:** identifiedBy: Kahanpää, Jere; dateIdentified: 09/26/2013; **Event:** year: 1947; month: 7; day: 14; habitat: coastal meadows; **Record Level:** institutionCode: MZH; basisOfRecord: PreservedSpecimen
- **Type status:** Other material. **Occurrence:** catalogNumber: http://id.luomus.fi/GV.16466; recordNumber: 606; recordedBy: Frey, Richard; individualCount: 1; sex: F; lifeStage: adult; **Taxon:** scientificName: *Coenia vulgata* Krivosheina, 2001; order: Diptera; family: Ephydridae; genus: Coenia; specificEpithet: vulgata; scientificNameAuthorship: Krivosheina, 2001; **Location:** country: Finland; stateProvince: PPe; municipality: Hailuoto; locality: Kirkkolahti; decimalLatitude: 64.98; decimalLongitude: 24.72; geodeticDatum: wgs84; coordinateUncertaintyInMeters: 2000; georeferencedBy: Kahanpää, Jere; **Identification:** identifiedBy: Kahanpää, Jere; dateIdentified: 09/26/2013; **Event:** year: 1947; month: 7; day: 12; habitat: coastal meadows, on water; **Record Level:** institutionCode: MZH; basisOfRecord: PreservedSpecimen
- **Type status:** Other material. **Occurrence:** catalogNumber: http://id.luomus.fi/GV.16467; recordNumber: 2557; recordedBy: Frey, Richard; individualCount: 1; sex: F; lifeStage: adult; preparations: gen. prep. on pin; **Taxon:** scientificName: *Coenia vulgata* Krivosheina, 2001; order: Diptera; family: Ephydridae; genus: Coenia; specificEpithet: vulgata; scientificNameAuthorship: Krivosheina, 2001; **Location:** country: Finland; stateProvince: PPe; municipality: Hailuoto; locality: Marjaniemi; decimalLatitude: 65.04; decimalLongitude: 24.56; geodeticDatum: wgs84; coordinateUncertaintyInMeters: 1000; georeferencedBy: Kahanpää, Jere; **Identification:** identifiedBy: Kahanpää, Jere; dateIdentified: 09/26/2013; **Event:** year: 1947; month: 7; day: 13; habitat: a coastal bay and a coastal grazing area; **Record Level:** institutionCode: MZH; basisOfRecord: PreservedSpecimen

#### Distribution

First recorded from Finland by Zatwarnicki and Kahanpää (2014). Previously known from Archangelsk Oblast (NW European Russia) and Kazakhstan (Krivosheina 2001).

#### Notes

Paratype specimen http://id.luomus.fi/GV.4185 is illustrated in Fig. 4a, c.

### Scatophila iowana

Wheeler, 1961

#### Materials

- **Type status:** Other material. **Occurrence:** catalogNumber: http://id.luomus.fi/HT.3120; recordedBy: Storå, Ragnar; individualCount: 1; sex: M; lifeStage: adult; **Taxon:** scientificName: *Scatophila iowana* Wheeler, 1961; order: Diptera; family: Ephydridae; genus: Scatophila; specificEpithet: iowana; scientificNameAuthorship: Wheeler, 1961; **Location:** country: Finland; stateProvince: KP; municipality: Pietarsaari; verbatimLocality: Fennia Om Jakobstad; decimalLatitude: 63.69; decimalLongitude: 22.67; geodeticDatum: WGS84; coordinateUncertaintyInMeters: 10000; georeferencedBy: Jere Kahanpää (MZH); **Identification:** identificationRemarks: Identified as Scatophila modesta Becker by MG Krivosheina in 2003.; **Event:** year: 1950; month: 7; day: 5; **Record Level:** institutionCode: MZH; basisOfRecord: PreservedSpecimen

#### Diagnosis

*Scatophila iowana* Wheeler has entirely black legs, a yellow haltere, a matt brown mesonotum and a brownish microtomentose abdomen. It lacks the pair of pronounced facial setae typical for *S. despecta* (Haliday). The wing and mesonotal patterns of *Scatophila iowana* resemble those of *S. caviceps* (Stenhammar). Males of these two species can be identified by the shape of the central part of the face: somewhat protruding between well-defined antennal grooves in *S. iowana*, weakly concave and without antennal grooves in *S. caviceps*.

The anepisternum of *S. iowana* is rather uniformly brownish; *S. caviceps* has a grey anepisternum with two brown spots, one around the base of the anepisternal bristle and one at the ventral margin.

The male terminalia of *S. iowana* are characterized by the anterior margin of the epandrium, which is slightly concave, and the aedeagus, which consists of three sclerites. In *S. caviceps* the anterior margin of the epandrium bears a medial projection and the aedeagus is a one-piece structure.

#### Distribution

First recorded from Finland by Zatwarnicki and Kahanpää (2014). *Scatophila iowana* is a Holarctic species. It is probably widespread in Europe, but its distribution remains poorly known (Zatwarnicki 2013).

### Scatophila mesogramma

(Loew, 1869)

#### Materials

- **Type status:** Other material. **Occurrence:** catalogNumber: http://id.luomus.fi/GV.19175; recordNumber: 6405; recordedBy: Storå, Ragnar; individualCount: 1; lifeStage: adult; **Taxon:** scientificName: *Scatophila mesogramma* (Loew, 1869); order: Diptera; family: Ephydridae; genus: Scatophila; specificEpithet: mesogramma; scientificNameAuthorship: (Loew, 1869); **Location:** country: Finland; stateProvince: KP; municipality: Pietarsaari; verbatimLocality: Jakobstad; decimalLatitude: 63.704; decimalLongitude: 22.634; geodeticDatum: wgs84; georeferencedBy: Kahanpää, Jere; **Identification:** identifiedBy: Kahanpää, Jere; dateIdentified: 2013-9-26; **Event:** year: 1954; month: 7; day: 27; **Record Level:** institutionCode: MZH; basisOfRecord: PreservedSpecimen
- **Type status:** Other material. **Occurrence:** catalogNumber: http://id.luomus.fi/GV.19176; recordNumber: 1743; recordedBy: Storå, Ragnar; individualCount: 1; lifeStage: adult; **Taxon:** scientificName: *Scatophila mesogramma* (Loew, 1869); order: Diptera; family: Ephydridae; genus: Scatophila; specificEpithet: mesogramma; scientificNameAuthorship: (Loew, 1869); **Location:** country: Finland; stateProvince: KP; municipality: Uusikaarlepyy; verbatimLocality: Nykarleby; decimalLatitude: 63.44; decimalLongitude: 22.675; geodeticDatum: wgs84; georeferencedBy: Kahanpää, Jere; **Identification:** identifiedBy: Kahanpää, Jere; dateIdentified: 2013-9-26; **Event:** year: 1955; month: 7; day: 20; **Record Level:** institutionCode: MZH; basisOfRecord: PreservedSpecimen
- **Type status:** Other material. **Occurrence:** catalogNumber: http://id.luomus.fi/GV.19177; recordNumber: 7133; recordedBy: Storå, Ragnar; individualCount: 1; lifeStage: adult; **Taxon:** scientificName: *Scatophila mesogramma* (Loew, 1869); order: Diptera; family: Ephydridae; genus: Scatophila; specificEpithet: mesogramma; scientificNameAuthorship: (Loew, 1869); **Location:** country: Finland; stateProvince: KP; municipality: Uusikaarlepyy; verbatimLocality: Nykarleby; decimalLatitude: 63.436; decimalLongitude: 22.675; geodeticDatum: wgs84; georeferencedBy: Kahanpää, Jere; **Identification:** identifiedBy: Kahanpää, Jere; dateIdentified: 2013-9-26; **Event:** year: 1955; month: 7; day: 27; **Record Level:** institutionCode: MZH; basisOfRecord: PreservedSpecimen
- **Type status:** Other material. **Occurrence:** catalogNumber: http://id.luomus.fi/GV.19178; recordNumber: 885; recordedBy: Frey, Richard; individualCount: 1; lifeStage: adult; **Taxon:** scientificName: *Scatophila mesogramma* (Loew, 1869); order: Diptera; family: Ephydridae; genus: Scatophila; specificEpithet: mesogramma; scientificNameAuthorship: (Loew, 1869); **Location:** country: Russia; stateProvince: Republic of Carelia; municipality: Paanajärvi; locality: behind Takalo farm; verbatimLocality: Paanajärvi; decimalLatitude: 66.26; decimalLongitude: 29.78; geodeticDatum: wgs84; georeferencedBy: Kahanpää, Jere; **Identification:** identifiedBy: Kahanpää, Jere; dateIdentified: 2013-9-26; **Event:** year: 1939; month: 6; day: 17; habitat: lake shore; **Record Level:** institutionCode: MZH; basisOfRecord: PreservedSpecimen
- **Type status:** Other material. **Occurrence:** catalogNumber: http://id.luomus.fi/GV.19179; recordNumber: 2005; recordedBy: Storå, Ragnar; individualCount: 1; lifeStage: adult; **Taxon:** scientificName: *Scatophila mesogramma* (Loew, 1869); order: Diptera; family: Ephydridae; genus: Scatophila; specificEpithet: mesogramma; scientificNameAuthorship: (Loew, 1869); **Location:** country: Finland; stateProvince: KP; municipality: Pietarsaari; verbatimLocality: Jakobstad; decimalLatitude: 63.704; decimalLongitude: 22.634; geodeticDatum: wgs84; georeferencedBy: Kahanpää, Jere; **Identification:** identifiedBy: Kahanpää, Jere; dateIdentified: 2013-9-26; **Event:** year: 1952; month: 8; day: 24; **Record Level:** institutionCode: MZH; basisOfRecord: PreservedSpecimen
- **Type status:** Other material. **Occurrence:** catalogNumber: http://id.luomus.fi/GV.19180; recordNumber: 2171; recordedBy: Storå, Ragnar; individualCount: 1; lifeStage: adult; **Taxon:** scientificName: *Scatophila mesogramma* (Loew, 1869); order: Diptera; family: Ephydridae; genus: Scatophila; specificEpithet: mesogramma; scientificNameAuthorship: (Loew, 1869); **Location:** country: Finland; stateProvince: KP; municipality: Pietarsaari; verbatimLocality: Jakobstad; decimalLatitude: 63.704; decimalLongitude: 22.634; geodeticDatum: wgs84; georeferencedBy: Kahanpää, Jere; **Identification:** identifiedBy: Kahanpää, Jere; dateIdentified: 2013-9-26; **Event:** year: 1952; month: 8; day: 30; **Record Level:** institutionCode: MZH; basisOfRecord: PreservedSpecimen
- **Type status:** Other material. **Occurrence:** catalogNumber: http://id.luomus.fi/GV.19181; recordNumber: 4726; recordedBy: Storå, Ragnar; individualCount: 1; lifeStage: adult; **Taxon:** scientificName: *Scatophila mesogramma* (Loew, 1869); order: Diptera; family: Ephydridae; genus: Scatophila; specificEpithet: mesogramma; scientificNameAuthorship: (Loew, 1869); **Location:** country: Finland; stateProvince: KP; municipality: Uusikaarlepyy; verbatimLocality: Nykarleby; decimalLatitude: 63.436; decimalLongitude: 22.675; geodeticDatum: wgs84; georeferencedBy: Kahanpää, Jere; **Identification:** identifiedBy: Kahanpää, Jere; dateIdentified: 2013-9-26; **Event:** year: 1954; month: 5; day: 24; **Record Level:** institutionCode: MZH; basisOfRecord: PreservedSpecimen
- **Type status:** Other material. **Occurrence:** catalogNumber: http://id.luomus.fi/GV.19182; recordNumber: 2215; recordedBy: Storå, Ragnar; individualCount: 1; lifeStage: adult; **Taxon:** scientificName: *Scatophila mesogramma* (Loew, 1869); order: Diptera; family: Ephydridae; genus: Scatophila; specificEpithet: mesogramma; scientificNameAuthorship: (Loew, 1869); **Location:** country: Finland; stateProvince: KP; municipality: Pietarsaari; verbatimLocality: Jakobstad; decimalLatitude: 63.704; decimalLongitude: 22.634; geodeticDatum: wgs84; georeferencedBy: Kahanpää, Jere; **Identification:** identifiedBy: Kahanpää, Jere; dateIdentified: 2013-9-26; **Event:** year: 1952; month: 8; day: 30; **Record Level:** institutionCode: MZH; basisOfRecord: PreservedSpecimen
- **Type status:** Other material. **Occurrence:** catalogNumber: http://id.luomus.fi/GV.19183; recordNumber: 7287; recordedBy: Storå, Ragnar; individualCount: 1; lifeStage: adult; **Taxon:** scientificName: *Scatophila mesogramma* (Loew, 1869); order: Diptera; family: Ephydridae; genus: Scatophila; specificEpithet: mesogramma; scientificNameAuthorship: (Loew, 1869); **Location:** country: Finland; stateProvince: KP; municipality: Uusikaarlepyy; verbatimLocality: Nykarleby; decimalLatitude: 63.436; decimalLongitude: 22.675; geodeticDatum: wgs84; georeferencedBy: Kahanpää, Jere; **Identification:** identifiedBy: Kahanpää, Jere; dateIdentified: 2013-9-26; **Event:** year: 1955; month: 8; day: 1; **Record Level:** institutionCode: MZH; basisOfRecord: PreservedSpecimen
- **Type status:** Other material. **Occurrence:** catalogNumber: http://id.luomus.fi/GV.19184; recordNumber: 4557; recordedBy: Storå, Ragnar; individualCount: 1; lifeStage: adult; **Taxon:** scientificName: *Scatophila mesogramma* (Loew, 1869); order: Diptera; family: Ephydridae; genus: Scatophila; specificEpithet: mesogramma; scientificNameAuthorship: (Loew, 1869); **Location:** country: Finland; stateProvince: KP; municipality: Pietarsaari; verbatimLocality: Jakobstad; decimalLatitude: 63.704; decimalLongitude: 22.634; geodeticDatum: wgs84; georeferencedBy: Kahanpää, Jere; **Identification:** identifiedBy: Kahanpää, Jere; dateIdentified: 2013-9-26; **Event:** year: 1954; month: 8; day: 15; **Record Level:** institutionCode: MZH; basisOfRecord: PreservedSpecimen
- **Type status:** Other material. **Occurrence:** catalogNumber: http://id.luomus.fi/GV.19185; recordNumber: 4559; recordedBy: Storå, Ragnar; individualCount: 1; lifeStage: adult; **Taxon:** scientificName: *Scatophila mesogramma* (Loew, 1869); order: Diptera; family: Ephydridae; genus: Scatophila; specificEpithet: mesogramma; scientificNameAuthorship: (Loew, 1869); **Location:** country: Finland; stateProvince: KP; municipality: Pietarsaari; verbatimLocality: Jakobstad; decimalLatitude: 63.704; decimalLongitude: 22.634; geodeticDatum: wgs84; georeferencedBy: Kahanpää, Jere; **Identification:** identifiedBy: Kahanpää, Jere; dateIdentified: 2013-9-26; **Event:** year: 1954; month: 8; day: 15; **Record Level:** institutionCode: MZH; basisOfRecord: PreservedSpecimen
- **Type status:** Other material. **Occurrence:** catalogNumber: http://id.luomus.fi/GV.19186; recordNumber: 4864; recordedBy: Storå, Ragnar; individualCount: 1; lifeStage: adult; **Taxon:** scientificName: *Scatophila mesogramma* (Loew, 1869); order: Diptera; family: Ephydridae; genus: Scatophila; specificEpithet: mesogramma; scientificNameAuthorship: (Loew, 1869); **Location:** country: Finland; stateProvince: KP; municipality: Uusikaarlepyy; verbatimLocality: Nykarleby; decimalLatitude: 63.436; decimalLongitude: 22.675; geodeticDatum: wgs84; georeferencedBy: Kahanpää, Jere; **Identification:** identifiedBy: Kahanpää, Jere; dateIdentified: 2013-9-26; **Event:** year: 1954; month: 5; day: 26; **Record Level:** institutionCode: MZH; basisOfRecord: PreservedSpecimen
- **Type status:** Other material. **Occurrence:** catalogNumber: http://id.luomus.fi/GV.19187; recordNumber: 1308; recordedBy: Frey, Richard; individualCount: 1; lifeStage: adult; **Taxon:** scientificName: *Scatophila mesogramma* (Loew, 1869); order: Diptera; family: Ephydridae; genus: Scatophila; specificEpithet: mesogramma; scientificNameAuthorship: (Loew, 1869); **Location:** country: Finland; stateProvince: InL; municipality: Utsjoki; verbatimLocality: Utsjoki; decimalLatitude: 69.509; decimalLongitude: 27.109; geodeticDatum: wgs84; georeferencedBy: Kahanpää, Jere; **Identification:** identifiedBy: Kahanpää, Jere; dateIdentified: 2013-9-26; **Record Level:** institutionCode: MZH; basisOfRecord: PreservedSpecimen
- **Type status:** Other material. **Occurrence:** catalogNumber: http://id.luomus.fi/GV.19188; recordNumber: 2041; recordedBy: Storå, Ragnar; individualCount: 1; lifeStage: adult; **Taxon:** scientificName: *Scatophila mesogramma* (Loew, 1869); order: Diptera; family: Ephydridae; genus: Scatophila; specificEpithet: mesogramma; scientificNameAuthorship: (Loew, 1869); **Location:** country: Finland; stateProvince: KP; municipality: Pietarsaari; verbatimLocality: Jakobstad; decimalLatitude: 63.704; decimalLongitude: 22.634; geodeticDatum: wgs84; georeferencedBy: Kahanpää, Jere; **Identification:** identifiedBy: Kahanpää, Jere; dateIdentified: 2013-9-26; **Event:** year: 1952; month: 8; day: 24; **Record Level:** institutionCode: MZH; basisOfRecord: PreservedSpecimen
- **Type status:** Other material. **Occurrence:** catalogNumber: http://id.luomus.fi/GV.19189; recordNumber: 4859; recordedBy: Storå, Ragnar; individualCount: 1; lifeStage: adult; **Taxon:** scientificName: *Scatophila mesogramma* (Loew, 1869); order: Diptera; family: Ephydridae; genus: Scatophila; specificEpithet: mesogramma; scientificNameAuthorship: (Loew, 1869); **Location:** country: Finland; stateProvince: KP; municipality: Uusikaarlepyy; verbatimLocality: Nykarleby; decimalLatitude: 63.436; decimalLongitude: 22.675; geodeticDatum: wgs84; georeferencedBy: Kahanpää, Jere; **Identification:** identifiedBy: Kahanpää, Jere; dateIdentified: 2013-9-26; **Event:** year: 1954; month: 5; day: 26; **Record Level:** institutionCode: MZH; basisOfRecord: PreservedSpecimen
- **Type status:** Other material. **Occurrence:** catalogNumber: http://id.luomus.fi/GV.19190; recordNumber: 4858; recordedBy: Storå, Ragnar; individualCount: 1; lifeStage: adult; **Taxon:** scientificName: *Scatophila mesogramma* (Loew, 1869); order: Diptera; family: Ephydridae; genus: Scatophila; specificEpithet: mesogramma; scientificNameAuthorship: (Loew, 1869); **Location:** country: Finland; stateProvince: KP; municipality: Uusikaarlepyy; verbatimLocality: Nykarleby; decimalLatitude: 63.436; decimalLongitude: 22.675; geodeticDatum: wgs84; georeferencedBy: Kahanpää, Jere; **Identification:** identifiedBy: Kahanpää, Jere; dateIdentified: 2013-9-26; **Event:** year: 1954; month: 5; day: 26; **Record Level:** institutionCode: MZH; basisOfRecord: PreservedSpecimen
- **Type status:** Other material. **Occurrence:** catalogNumber: http://id.luomus.fi/GV.19191; recordNumber: 4856; recordedBy: Storå, Ragnar; individualCount: 1; lifeStage: adult; **Taxon:** scientificName: *Scatophila mesogramma* (Loew, 1869); order: Diptera; family: Ephydridae; genus: Scatophila; specificEpithet: mesogramma; scientificNameAuthorship: (Loew, 1869); **Location:** country: Finland; stateProvince: KP; municipality: Uusikaarlepyy; verbatimLocality: Nykarleby; decimalLatitude: 63.436; decimalLongitude: 22.675; geodeticDatum: wgs84; georeferencedBy: Kahanpää, Jere; **Identification:** identifiedBy: Kahanpää, Jere; dateIdentified: 2013-9-26; **Event:** year: 1954; month: 5; day: 26; **Record Level:** institutionCode: MZH; basisOfRecord: PreservedSpecimen
- **Type status:** Other material. **Occurrence:** catalogNumber: http://id.luomus.fi/GV.19192; recordNumber: 408; recordedBy: Hellén, Wolter; individualCount: 1; lifeStage: adult; preparations: gen. prep. on pin; **Taxon:** scientificName: *Scatophila mesogramma* (Loew, 1869); order: Diptera; family: Ephydridae; genus: Scatophila; specificEpithet: mesogramma; scientificNameAuthorship: (Loew, 1869); **Location:** country: Russia; stateProvince: Murmansk Oblast; verbatimLocality: Kola; decimalLatitude: 68.88; decimalLongitude: 33.04; geodeticDatum: wgs84; georeferencedBy: Kahanpää, Jere; **Identification:** identifiedBy: Kahanpää, Jere; dateIdentified: 2013-9-26; **Event:** year: 1913; month: 7; day: 29; **Record Level:** institutionCode: MZH; basisOfRecord: PreservedSpecimen
- **Type status:** Other material. **Occurrence:** catalogNumber: http://id.luomus.fi/GV.19193; recordNumber: 3871; recordedBy: Frey, Richard; individualCount: 1; lifeStage: adult; **Taxon:** scientificName: *Scatophila mesogramma* (Loew, 1869); order: Diptera; family: Ephydridae; genus: Scatophila; specificEpithet: mesogramma; scientificNameAuthorship: (Loew, 1869); **Location:** country: Finland; stateProvince: EnL; municipality: Enontekiö; locality: Palojoensuu; verbatimLocality: Enontekis; decimalLatitude: 68.296; decimalLongitude: 22.983; geodeticDatum: wgs84; georeferencedBy: Kahanpää, Jere; **Identification:** identifiedBy: Kahanpää, Jere; dateIdentified: 2013-9-26; **Event:** year: 1911; month: 7; day: 11; **Record Level:** institutionCode: MZH; basisOfRecord: PreservedSpecimen
- **Type status:** Other material. **Occurrence:** catalogNumber: http://id.luomus.fi/GV.19194; recordNumber: 4397; recordedBy: Frey, Richard; individualCount: 1; lifeStage: adult; **Taxon:** scientificName: *Scatophila mesogramma* (Loew, 1869); order: Diptera; family: Ephydridae; genus: Scatophila; specificEpithet: mesogramma; scientificNameAuthorship: (Loew, 1869); **Location:** country: Finland; stateProvince: EnL; municipality: Enontekiö; verbatimLocality: Enontekis; decimalLatitude: 68.379; decimalLongitude: 22.725; geodeticDatum: wgs84; georeferencedBy: Kahanpää, Jere; **Identification:** identifiedBy: Kahanpää, Jere; dateIdentified: 2013-9-26; **Event:** year: 1911; month: 7; day: 14; **Record Level:** institutionCode: MZH; basisOfRecord: PreservedSpecimen
- **Type status:** Other material. **Occurrence:** catalogNumber: http://id.luomus.fi/GV.19195; recordNumber: 2212; recordedBy: Storå, Ragnar; individualCount: 1; lifeStage: adult; **Taxon:** scientificName: *Scatophila mesogramma* (Loew, 1869); order: Diptera; family: Ephydridae; genus: Scatophila; specificEpithet: mesogramma; scientificNameAuthorship: (Loew, 1869); **Location:** country: Finland; stateProvince: KP; municipality: Pietarsaari; verbatimLocality: Jakobstad; decimalLatitude: 63.704; decimalLongitude: 22.634; geodeticDatum: wgs84; georeferencedBy: Kahanpää, Jere; **Identification:** identifiedBy: Kahanpää, Jere; dateIdentified: 2013-9-26; **Event:** year: 1952; month: 8; day: 20; **Record Level:** institutionCode: MZH; basisOfRecord: PreservedSpecimen

#### Distribution

New to Finland and Russia. First recorded from Finland by Zatwarnicki and Kahanpää (2014). Holarctic; in Europe it is known from Finland, Sweden, Denmark, Great Britain, Belgium and Germany (Zatwarnicki 2013).

## Discussion

### Notes on previous misidentifications

The species identified as *Discocerina pulicaria* [auct. nec Haliday] by Frey (1933), Frey (1937), Frey (1949), Lindberg and Saris (1952) is actually *Ditrichophora fuscella* (Stenhammar, 1844). Similarly, *Allotrichoma laterale* sensu Frey (1940b), Frey (1940a) is *A. bezzii* Becker, 1896. Previous records of *Philygria posticata* by Frey 1930 represent *Ph. femorata* (Stenhammar).

## Supplementary Material

XML Treatment for Trimerina microchaeta

XML Treatment for Hydrellia albifrons

XML Treatment for Hydrellia cochleariae

XML Treatment for Hydrellia fulviceps

XML Treatment for Hydrellia laticeps

XML Treatment for Hydrellia mutata

XML Treatment for Hydrellia subalbiceps

XML Treatment for Hydrellia tarsata

XML Treatment for Allotrichoma bezzii

XML Treatment for Ditrichophora calceata

XML Treatment for Hecamedoides unispinosus

XML Treatment for Pelina norvegica

XML Treatment for Parydra (Chaetoapnea) mitis

XML Treatment for Calocoenia paurosoma

XML Treatment for Coenia vulgata

XML Treatment for Scatophila iowana

XML Treatment for Scatophila mesogramma

## Figures and Tables

**Figure 1a.**
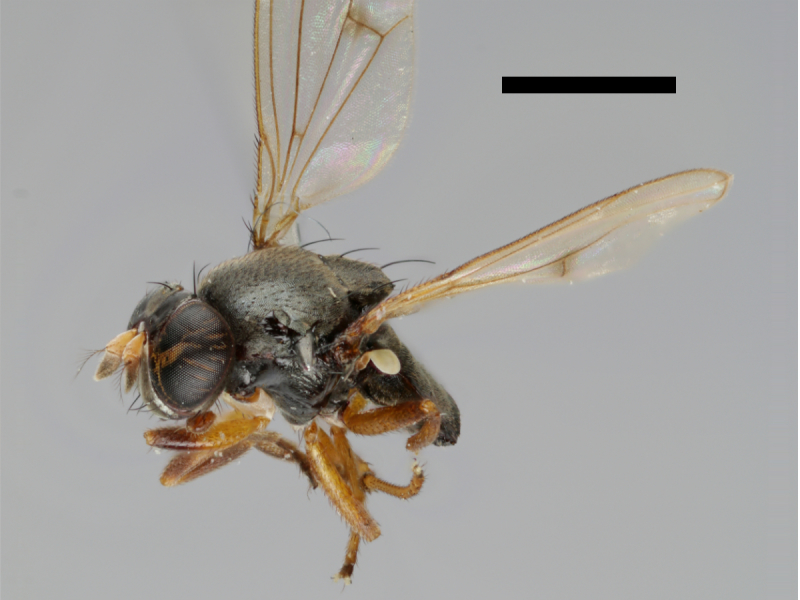
*Trimerina microchaeta*. Holotype of *Trimerina indistincta* Krivosheina (specimen http://id.luomus.fi/GV.4020)

**Figure 1b.**
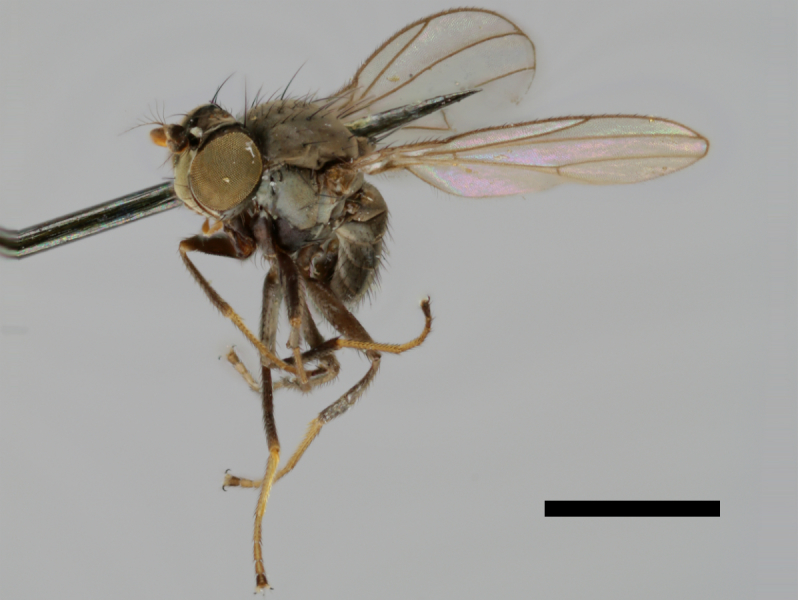
*Hydrellia mutata* (http://id.luomus.fi/GV.16611)

**Figure 1c.**
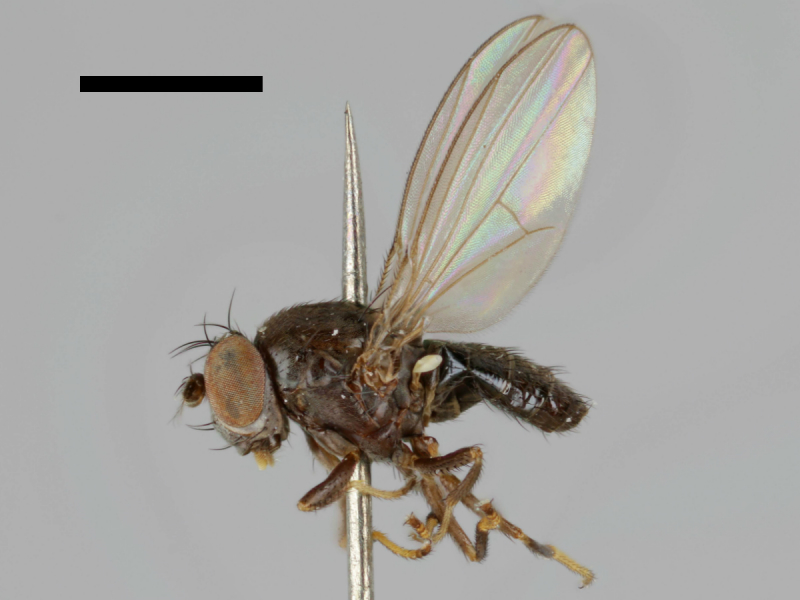
*Ditrichophora calceata* (http://id.luomus.fi/GV.16561)

**Figure 1d.**
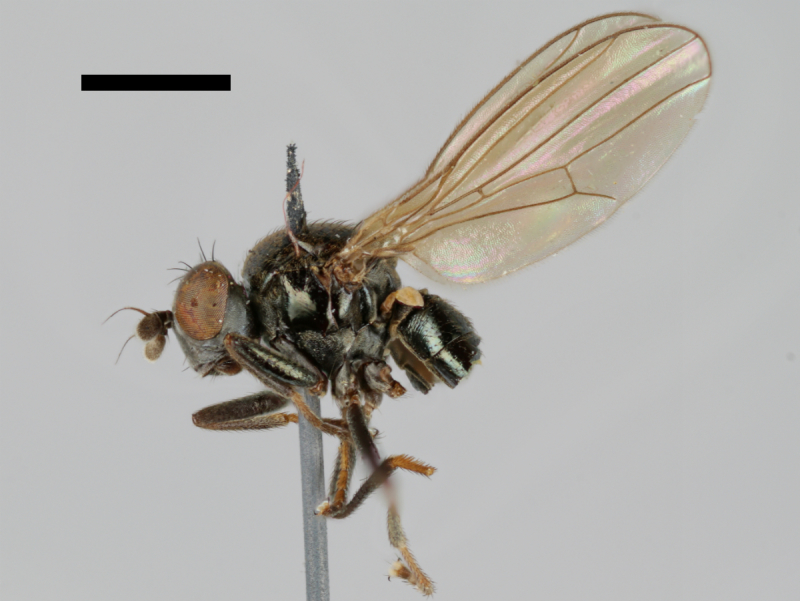
*Pelina norvegica* (http://id.luomus.fi/GV.19162)

**Figure 2a.**
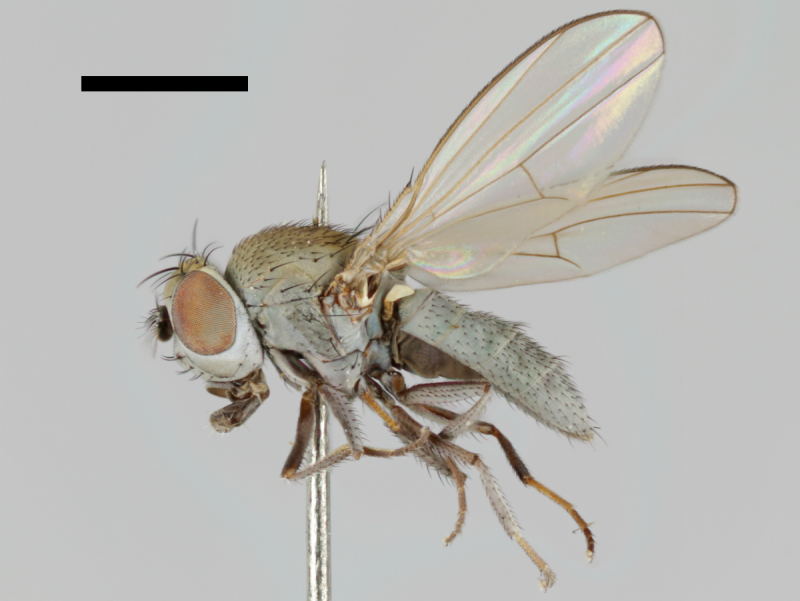
*Hecamedoides glaucellus* (specimen http://id.luomus.fi/GV.19146)

**Figure 2b.**
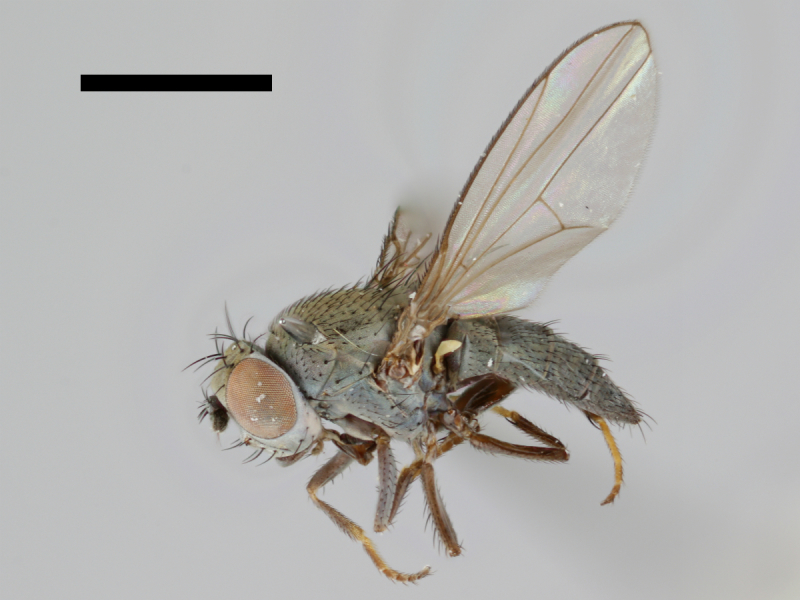
*Hecamedoides unispinosus* (specimen http://id.luomus.fi/GV.19147)

**Figure 3a.**
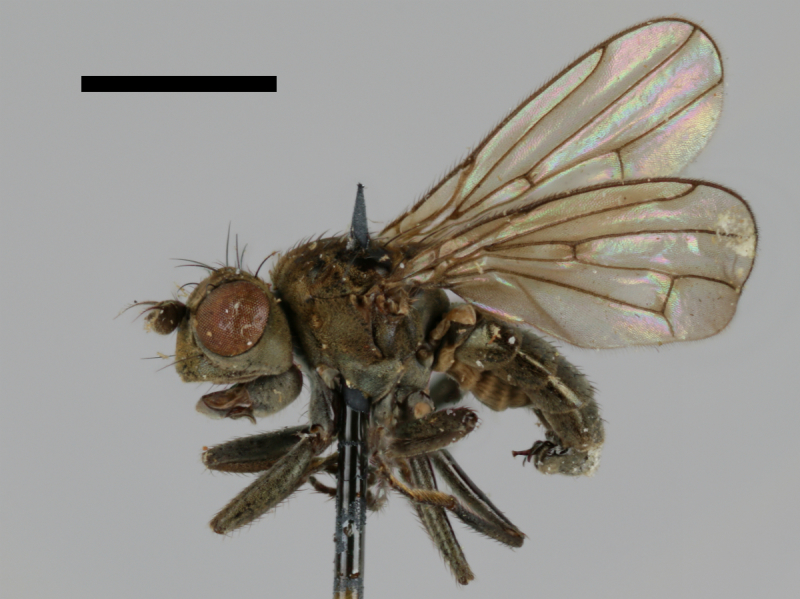
*Parydra arctica*, male (specimen http://id.luomus.fi/GV.16507)

**Figure 3b.**
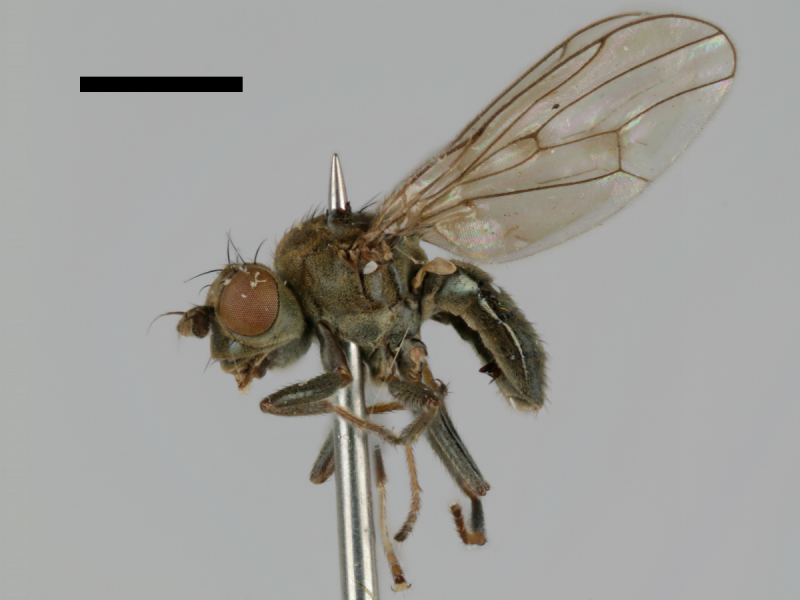
*Parydra fossarum*, male (specimen http://id.luomus.fi/GV.16512)

**Figure 3c.**
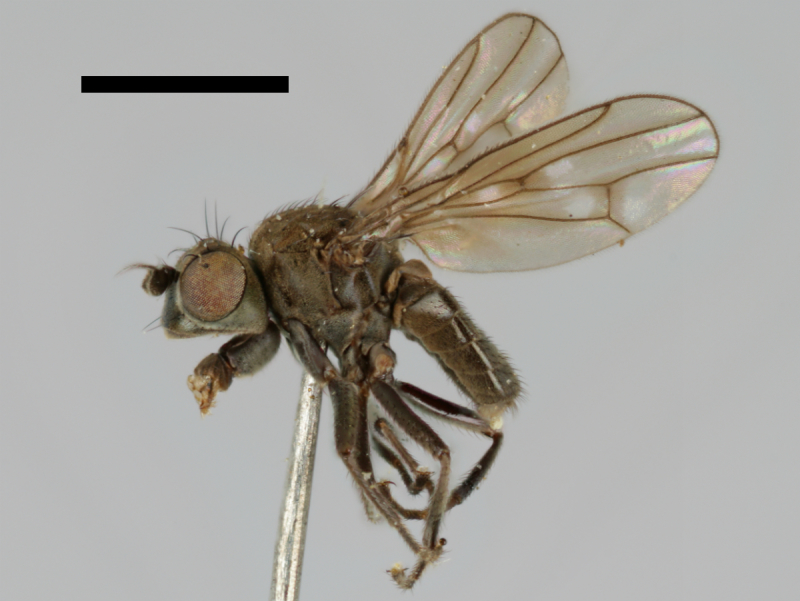
*Parydra mitis*, female (specimen http://id.luomus.fi/GV.16525)

**Figure 3d.**
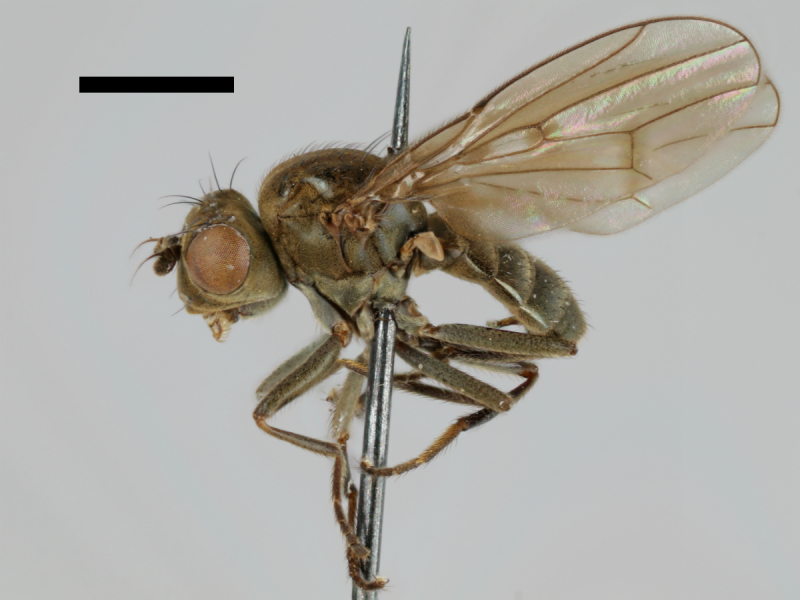
*Parydra nigritarsis*, male (specimen http://id.luomus.fi/GV.19145)

**Figure 4a.**
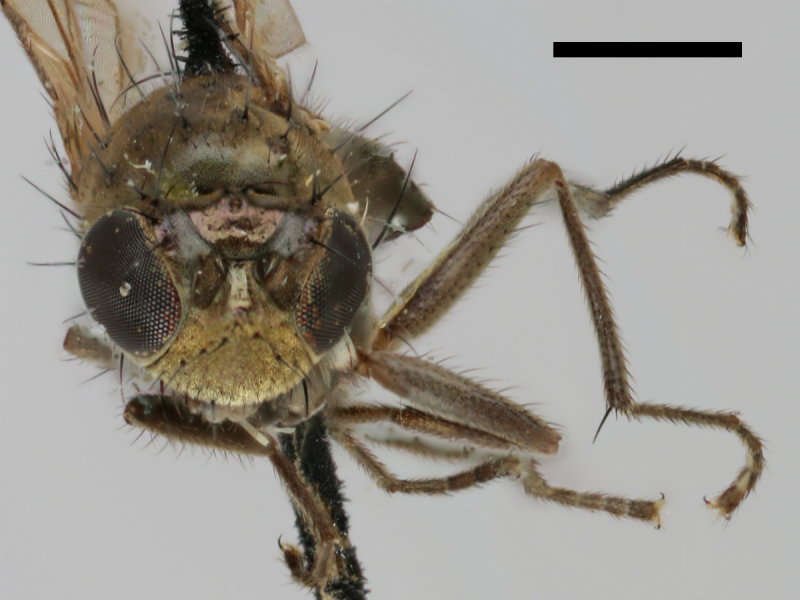
*Coenia vulgata*, face. Paratype specimen http://id.luomus.fi/GV.4185.

**Figure 4b.**
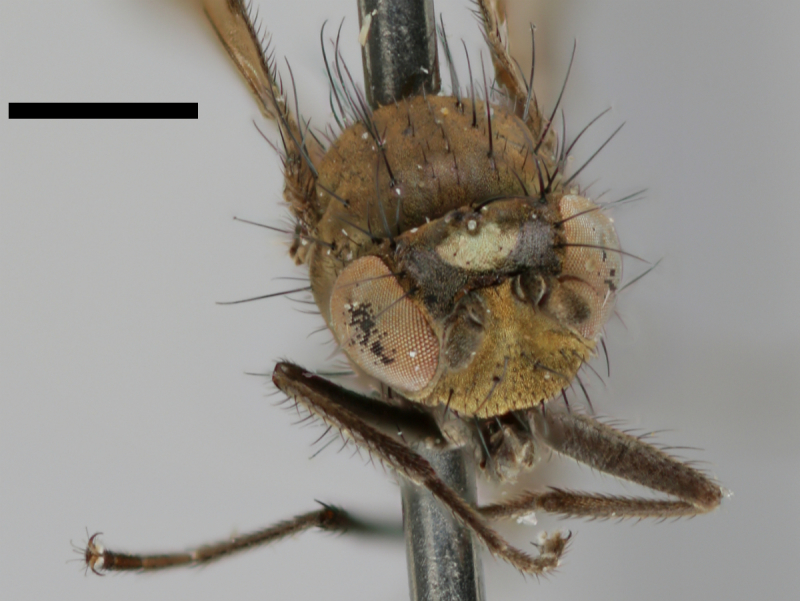
*Calocoenia paurosoma*, face. Specimen http://id.luomus.fi/GV.16455.

**Figure 4c.**
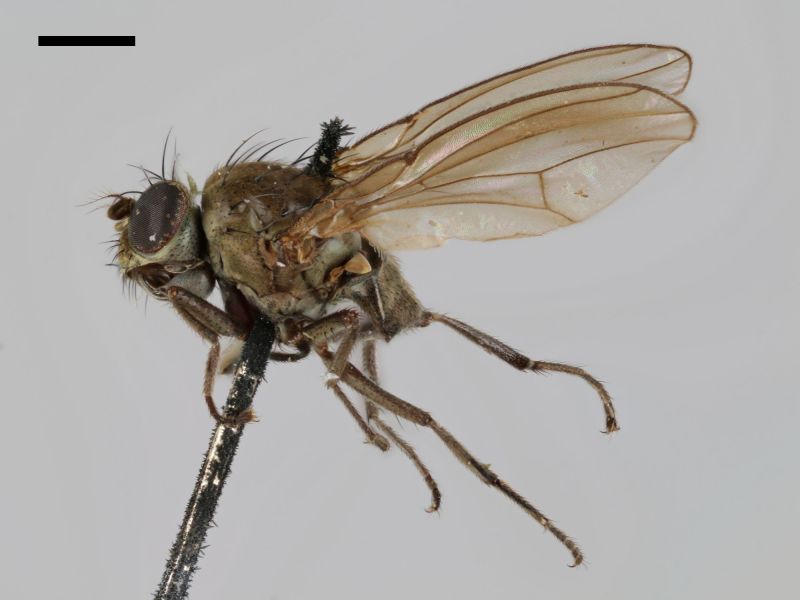
*Coenia vulgata*, lateral view. Specimen as above.

**Figure 4d.**
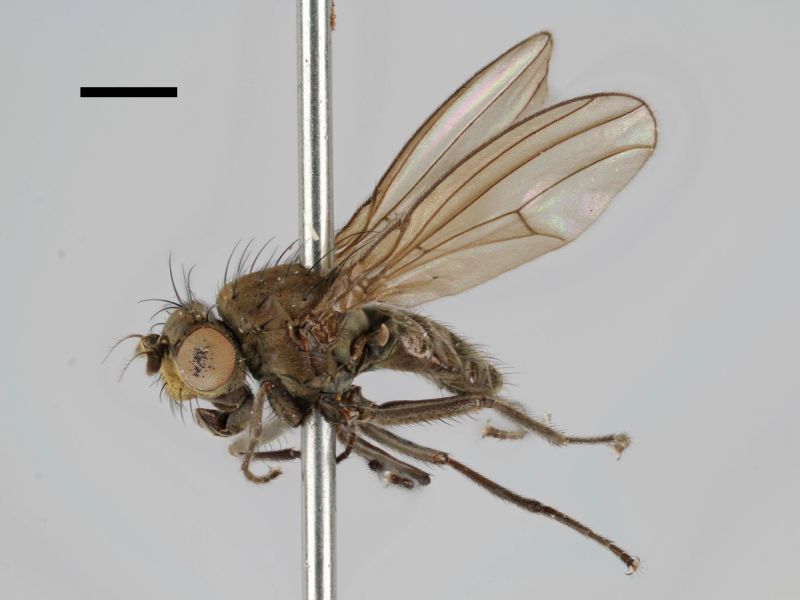
*Calocoenia paurosoma*, lateral view. Specimen as above.
